# Multiple Domains in the Rhizobial Type III Effector Bel2-5 Determine Symbiotic Efficiency With Soybean

**DOI:** 10.3389/fpls.2021.689064

**Published:** 2021-06-07

**Authors:** Safirah Tasa Nerves Ratu, Atsushi Hirata, Christian Oliver Kalaw, Michiko Yasuda, Mitsuaki Tabuchi, Shin Okazaki

**Affiliations:** ^1^United Graduate School of Agricultural Science, Tokyo University of Agriculture and Technology, Fuchu, Japan; ^2^Department of Applied Biological Science, Faculty of Agriculture, Kagawa University, Kagawa, Japan; ^3^Graduate School of Agriculture, Tokyo University of Agriculture and Technology, Fuchu, Japan

**Keywords:** symbiosis, nodulation, rhizobia, type III secretion system, effector, soybean

## Abstract

*Bradyrhizobium elkanii* utilizes the type III effector Bel2-5 for nodulation in host plants in the absence of Nod factors (NFs). In soybean plants carrying the *Rj4* allele, however, Bel2-5 causes restriction of nodulation by triggering immune responses. Bel2-5 shows similarity with XopD of the phytopathogen *Xanthomonas campestris* pv. *vesicatoria* and possesses two internal repeat sequences, two ethylene (ET)-responsive element-binding factor-associated amphiphilic repression (EAR) motifs, a nuclear localization signal (NLS), and a ubiquitin-like protease (ULP) domain, which are all conserved in XopD except for the repeat domains. By mutational analysis, we revealed that most of the putative domains/motifs in Bel2-5 were essential for both NF-independent nodulation and nodulation restriction in *Rj4* soybean. The expression of soybean symbiosis- and defense-related genes was also significantly altered by inoculation with the *bel2-5* domain/motif mutants compared with the expression upon inoculation with wild-type *B. elkanii*, which was mostly consistent with the phenotypic changes of nodulation in host plants. Notably, the functionality of Bel2-5 was mostly correlated with the growth inhibition effect of Bel2-5 expressed in yeast cells. The nodulation phenotypes of the domain-swapped mutants of Bel2-5 and XopD indicated that both the C-terminal ULP domain and upstream region are required for the Bel2-5-dependent nodulation phenotypes. These results suggest that Bel2-5 interacts with and modifies host targets via these multiple domains to execute both NF-independent symbiosis and nodulation restriction in *Rj4* soybean.

## Introduction

Legume plants cope with nitrogen-deficient soils by establishing symbiosis with nitrogen-fixing bacteria, called rhizobia. This symbiosis results in the formation of a unique organ, the root nodule, where rhizobia provide ammonia for plants in exchange for carbon sources and other nutrients essential for their growth (Oldroyd et al., 2011). This symbiosis is of agronomic importance, as it reduces the need for chemical fertilizers in the production of legume crops such as soybean (Soumare et al., 2020).

Root nodule symbiosis is a highly specific interaction, as each rhizobium defines its host legumes via molecular dialogs with the host. In the first step, the bacteria recognize root-derived flavonoids secreted by legumes. The host-derived flavonoids induce the expression of rhizobial nodulation (*nod*) genes, which leads to the biosynthesis and secretion of lipochitooligosaccharides, called Nod factors (NFs). Nod factors are perceived by host lysin-motif receptors (Nod factor receptors, NFRs), and the perception activates the nodulation signaling cascade, leading to bacterial infection and nodule organogenesis (Madsen et al., 2003; Radutoiu et al., 2003). In addition to NFs, rhizobia have evolved several strategies to promote their infection of plants, including the use of surface polysaccharides such as extracellular polysaccharides, capsular polysaccharides, and lipopolysaccharides (Becker et al., 2005; Simsek et al., 2007; Kawaharada et al., 2015). However, most remarkably, numerous rhizobia utilize a type III secretion system (T3SS), which in pathogenic bacteria is known for its delivery of virulence factors, to promote symbiosis with host legumes (Deakin and Broughton, 2009).

Some gram-negative bacteria can deliver effector proteins from the bacterial cytoplasm into a eukaryotic host cell through a syringe-like multi-protein apparatus, the T3SS (Diepold and Wagner, 2014). Plant pathogens often use the T3SS to inject effector proteins (T3Es) into host cells to suppress the host immune response and promote infection (Coburn et al., 2007). To counteract these threats, plants have evolved to produce resistance (R) proteins that perceive the presence or action of effector proteins and allow them to elicit resistance responses called effector-triggered immunity (ETI), which halts pathogen invasion and disease (Jones and Dangl, 2006). Thus, bacterial T3Es and the plant immune system both contribute to shaping plant-pathogen interactions. Similarly, rhizobial T3Es often act as determinants of host specificity. Depending on the host cultivar (genotype), they might affect symbiosis either positively or negatively. Some rhizobia utilize T3Es to enhance nodule formation by suppressing host defense responses by modulating the MAPK cascade (Miwa and Okazaki, 2017). In soybean cultivars carrying *Rj* genes, recognition of particular rhizobial T3SS (and thus T3Es) restricts bacterial infection and nodule organogenesis (Okazaki et al., 2009; Faruque et al., 2015; Sugawara et al., 2018; Shobudani et al., 2020). This commonality between pathogenic bacteria and rhizobia suggests that they utilize similar genetic traits to invade and establish compatible association with plant hosts.

Recently, a distinct role of the rhizobial T3SS has been discovered in two different legume-rhizobium interactions: between *Bradyrhizobium elkanii* and soybean and between *Bradyrhizobium* sp. ORS3257 and an aquatic legume of the genus *Aeschynomene* (Okazaki et al., 2013, 2016). In these interactions, *Bradyrhizobium* strains utilize the T3SS to induce nodule formation in the absence of NF signals, which have been considered essential for nodule formation (Okazaki et al., 2013, 2016; Teulet et al., 2019). The T3Es responsible for the NF-independent pathway have also been identified; effector required for nodulation-A (ErnA) of *Bradyrhizobium* sp. ORS3257 (Teulet et al., 2019) and Bel2-5 of *B. elkanii* (Ratu et al., 2021). *Aeschynomene indica*, a host plant of *Bradyrhizobium* sp. ORS3257, is known for its nodulation, which does not require NFs. However, in ORS3257 a mutation of T3SS or *ernA* led to a Nod- phenotype (Teulet et al., 2019). Furthermore, ectopic expression of ErnA triggered cell division, resulting in the formation of nodule-like structures (Teulet et al., 2019). Bel2-5 of *B. elkanii* has been identified as a T3E that enables nodulation on the non-nodulating soybean En1282 (an *nfr1* mutant) (Ratu et al., 2021). In soybean roots, Bel2-5 could upregulate symbiosis-related genes and downregulate defense-related genes (Ratu et al., 2021). To our knowledge, Bel2-5 and ErnA have only a limited level of sequence similarity and are capable of inducing nodules only in their host legumes (e.g., soybean and *Aeschynomene*, respectively). These results suggest that rhizobia possibly customized specific T3SS and T3Es to reflect on the particular symbiotic partners.

Bel2-5 of *B. elkanii* resembles the XopD effector of the phytopathogen *Xanthomonas campestris* (*Xcv.*). While Bel2-5 can trigger nodulation signaling in the soybean *nfr1* mutant, the presence of Bel2-5 causes restriction of nodulation on soybean carrying the *Rj4* allele (Faruque et al., 2015; Ratu et al., 2021). *In silico* analysis showed that Bel2-5 possesses two internal repeat domains, two ethylene (ET)-responsive element-binding factor-associated amphiphilic repression (EAR) motifs, a nuclear localization signal (NLS), and a ubiquitin-like protease (ULP) domain (Ratu et al., 2021). Intriguingly, XopD also possesses all of these domains except for the repeat domains, and which play pivotal roles in pathogenicity in host plants (Hotson et al., 2003; Kim et al., 2008, 2013). However, the functions of these domains/motifs of Bel2-5 in symbiotic interactions remain unclear. In this study, we investigated the importance of the domains/motifs of the Bel2-5 effector in symbiosis in the context of both NF-independent nodulation in *nfr1* soybean and nodulation restriction in *Rj4* soybean.

## Materials and Methods

### Bacterial and Yeast Strains and Their Growth Conditions

The bacterial strains and plasmids used in this study are listed in Supplementary Table S1. *B. elkanii* strains were grown at 28°C in arabinose-gluconate (AG) (Sadowsky et al., 1987) medium supplemented with appropriate antibiotics (50 μg/mL polymyxin, 200 μg/mL kanamycin, 200 μg/mL streptomycin, and 200 μg/mL spectinomycin). *Escherichia coli* strains were grown at 37°C in Luria-Bertani medium (Green and Sambrook, 2012) supplemented with appropriate antibiotics (50 μg/mL kanamycin, 50 μg/mL streptomycin, and 50 μg/mL spectinomycin). Yeast strains were grown in yeast extract, peptone and dextrose (YPD) medium, synthetic dextrose (SD) medium (2% glucose, 0.67% yeast nitrogen base without amino acids) or synthetic galactose (SGal) medium (2% galactose, 0.67% yeast nitrogen base without amino acids). Appropriate amino acids and bases were added to the SD or SGal medium as necessary. Yeast cells were cultured at 26°C unless otherwise stated. All conjugation processes for *B. elkanii* mutant construction were performed on peptone-salts-yeast extract (PSY) medium (Regensburger and Hennecke, 1983).

### Plasmid Construction and Mutagenesis of *Bradyrhizobium elkanii*

All the constructs prepared in this study are listed in Supplementary Table S1, which includes the primers and cloning strategies. The construction of *B. elkanii* strains carrying mutated *bel2-5* derivatives (substitution or deletion) was performed using a QuikChange Lightning Site-Directed Mutagenesis Kit (Agilent Technologies, CA, United States). First, a ∼4.5-kb DNA fragment containing *bel2-5* with its promoter sequence (Supplementary Data S1) was synthesized (GENEWIZ, NJ, United States) and cloned into the carrier plasmid pUC57, which was then used as the template for generating plasmids carrying a substitution or deletion of targeted sequences using specific primer sets (Supplementary Table S1). The constructed substitution or deletion plasmids were confirmed by Sanger sequencing. Next, the *bel2-5* sequence with desired point mutations was excised from the carrier plasmid (pUC57) by digestion at the *Eco*RI/*Xba*I sites or further amplified to create *Sac*I/*Kpn*I sites for cloning. The resultant DNA fragments were transferred into the plasmid pK18mob or pBjGroEL4:dsRED and integrated into the *bel2-5* deletion mutant (*Δbel2-5*) through single-crossover recombination. The constructed *B. elkanii* mutant strains were screened by antibiotic resistance testing and confirmed by PCR and sequence analysis.

For swapping the ULP-like domain region between the Bel2-5 and XopD effectors, artificial sequences were synthesized as detailed in Supplementary Data S2 (GENEWIZ, NJ, United States) and cloned into the carrier plasmid pUC57. The synthetic sequences were then used as the template for PCR amplification using specific primer sets for cloning into the plasmid pBjGroEL4:dsRED at the *Sac*I/*Kpn*I sites. The constructed plasmids were confirmed using Sanger sequencing, then integrated into the *bel2-5* deletion mutant (*Δbel2-5*) through a single homologous recombination. The constructed *B. elkanii* mutant strains were screened by antibiotic resistance and confirmed by PCR.

### Nodulation Tests

The soybean cultivars used in this study were *Glycine max* (L.) Merr. En1282 (an *nfr1* mutant) and BARC-2 (*Rj4/Rj4*). Soybean seeds were surface-sterilized and germinated as previously described (Krause et al., 2002); 2-day-old germinated seedlings were transferred into a plant box (CUL-JAR300; Iwaki, Japan) containing sterilized vermiculite and inoculated with 1 × 10^7^ cells/mL *B. elkanii* strains or sterilized water (mock treatment). Plants were cultivated in a plant growth chamber at 25°C and 70% humidity under a 16/8-h day/night regimen; an appropriate amount of B&D nitrogen-free solution (Broughton and Dilworth, 1971) was added to water the plants. Nodule numbers were determined at 30 days after inoculation (dai).

### Expression of Bel2-5 and Its Mutated Derivatives in Yeast Cells

The full-length *bel2-5* sequence without a stop codon (∼4.0-kb) was amplified using a specific primer set according to the Gateway Technology Kit instruction manual (Invitrogen, CA, United States). The amplified PCR product was then used to generate a yeast-inducible expression plasmid as described previously (Tabuchi et al., 2009). Briefly, further PCR amplification was performed using *attB* adaptor primers to obtain the *att*B-flanked PCR product, which was cloned into the donor vector pDONR207 by the BP reaction. The entry clone was screened for antibiotic resistance and confirmed by PCR and sequence analysis. Next, the constructed entry clone of Bel2-5 was subcloned into the Gateway vector pMT751 via the LR reaction and then transformed into the yeast MTY914 (*smt3*Δ:*KanMX4* carrying pRS415-HA-*SMT3*). Yeast carrying the *bel2-5* sequence was used for localization and spot assays to check yeast growth inhibition. Partial deletion or substitution of *bel2-5* was performed by inverse PCR with specific primer sets using KOD-Plus-Neo DNA polymerase (Toyobo, Osaka, Japan). Inserts were sequenced to ensure that no mutations were introduced due to manipulation. For yeast growth inhibition assay, yeast cells carrying plasmids expressing Bel2-5 or its derivatives were suspended in sterilized water and the cell suspensions were measured and adjusted to OD_600__*nm*_ = 1.0. The cell suspensions were spotted into 10-fold serial dilution on SD (-ura) and SGal (-ura) plates and grown at 26°C for 2–3 days.

### Fluorescence Microscopy

Localization of Bel2-5 in yeast cells was performed as described previously (Tabuchi et al., 2009). Briefly, GFP fluorescence in non-fixed yeast cells was observed using an Olympus BX51 microscope (Olympus, Japan) with a GFP filter. Images were captured with a Hamamatsu C11440-10C Orca-Flash 2.8 CMOS camera (Hamamatsu Photonics, Japan) using Metamorph software (Molecular Devices, Japan).

### Soybean RNA Extraction and qRT-PCR Analysis

#### RNA Extraction

En1282 (*nfr1* mutant) and BARC-2 (*Rj4/Rj4*) soybean seeds were surface sterilized, germinated at 25°C for 2 days, and then transferred to a seed pack (Daiki Rika Co., Ltd., Japan) with B&D nitrogen-free solution. On the following day (the third day of germination), seeds were inoculated with 2 × 10^7^ cells/mL (for En1282 soybean) or 1 × 10^7^ cells/mL (for BARC-2 soybean) bacterial cultures. Then, at three or four dai, the soybean roots were immediately frozen in liquid nitrogen and then ground to a fine powder; 100 mg of the powder was used for total RNA extraction using an RNeasy Plant Mini Kit (Qiagen, Hilden, Germany) and treated with DNase I (Qiagen, Hilden, Germany) according to the manufacturer’s instructions. RNA quality and concentration were evaluated using a NanoDrop 2000/200c (Thermo Fisher, MA, United States).

#### qRT-PCR Analysis

The extracted RNA samples were used as the template for cDNA synthesis in a final volume of 20 μL (containing 400 ng of total RNA) by using the SuperScript III First-Strand Synthesis System for RT-PCR (Invitrogen, CA, United States). The RT-qPCR mixture and thermal cycling conditions were set as previously described (Yasuda et al., 2016). qRT-PCR was performed using a StepOne real-time PCR system (Applied Biosystems, MA, United States) with the primer set listed in Supplementary Table S1. Transcript levels of soybean genes were normalized to those of the housekeeping gene *SUBI-2* (Brechenmacher et al., 2008), which were measured in the same samples.

### Statistical Analysis

Data were analyzed using Student’s *t*-test and Tukey’s honestly significant difference (HSD) test, performed using IBM SPSS Statistics 22.0 software.

## Results

### Nodulation Phenotype of Soybean Infected With *bel2-5* Mutants

Bel2-5 shares similarity with the rhizobial T3E NopD (Xiang et al., 2020) and with XopD (Noël et al., 2002), from the plant pathogen *Xcv*. (Ratu et al., 2021). *In silico* analysis revealed that Bel2-5 possesses two repeat sequences (designated repeat domains 1 and 2), two EAR motifs, an NLS, and a ULP domain (Ratu et al., 2021, Figure 1).

**FIGURE 1:**
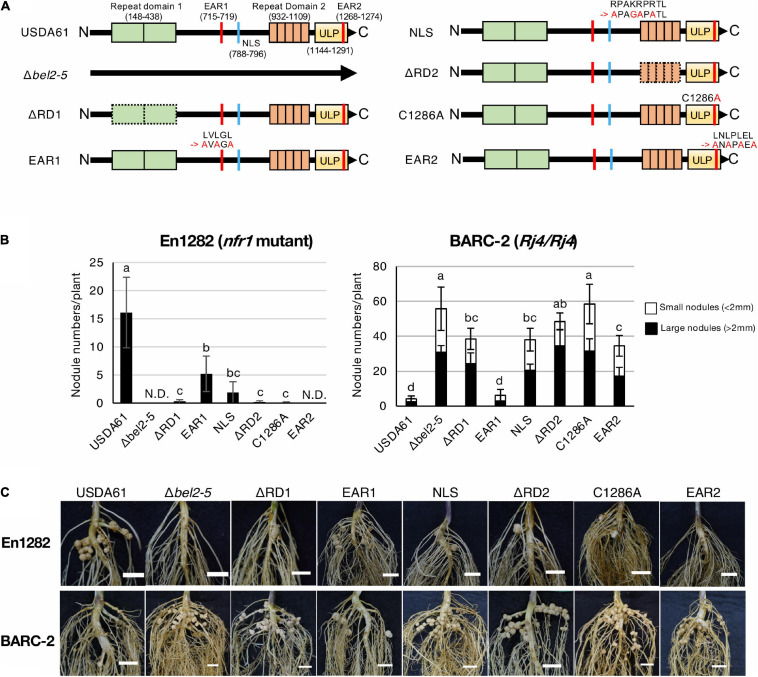
Nodulation phenotypes of *B. elkanii* USDA61 wild-type and the *bel2-5* deletion mutant and its derivatives (substitutions or deletions) on En1282 (*nfr1* mutant) and BARC-2 (*Rj4*/*Rj4*) soybean plants. **(A)** Schematic representation of Bel2-5 and its mutant derivatives in USDA61 and mutant strains. Bel2-5 possesses two internal repeat domains, two ethylene-responsive element-binding factor-associated amphiphilic repression (EAR) motifs, a nuclear localization signal (NLS), and a ubiquitin-like protease (ULP) domain. **(B)** Nodulation of *G. max* cv. En1282 and BARC-2 inoculated with wild-type and *bel2-5* mutant strains of *B. elkanii* USDA61. Nodule numbers were counted at 30 days after inoculation (dai). The values represent the mean ± SD (*n* = at least 10 plants, from two independent inoculation tests). N.D., not detected. Means followed by different letters are significantly different at the 0.05 level by Tukey’s test. **(C)** Roots of En1282 and BARC-2 inoculated with wild-type and mutant strains of *B. elkanii* USDA61. Roots were photographed at 30 dai. Scale bars, 1 cm. USDA61, *Bradyrhizobium elkanii* wild-type; Δ*bel2-5*, *bel2-5* knockout mutant; ΔRD1, repeat domain I deletion mutant; BERD1; EAR1, amino acid substitution mutant of the EAR1; BEEAR1; NLS, amino acid substitution mutant of the NLS; BENLS; ΔRD2, repeat domain 2 deletion mutant; BERD2; C1286A, amino acid substitution mutant of the ULP domain; BEC1286A; and EAR2, amino acid substitution mutant of the EAR2; BEEAR2.

To elucidate the function of each domain/motif in Bel2-5 during symbiosis with soybean, we deleted or substituted the region or critical amino acids in the predicted domain/motif (Figure 1A). The constructed mutants as well as the wild type (WT) and the *bel2-5* deletion mutant (Δ*bel2-5*) of *B. elkanii* strains were inoculated on the soybean cultivars En1282 (*nfr1* mutant) and BARC-2 (*Rj4/Rj4*). In En1282, nodulation was observed in roots inoculated with USDA61, whereas nodulation was completely abolished following inoculation with the *bel2-5* deletion mutant (Figures 1B,C). The nodule numbers observed with the other mutants, namely, BERD1, BERD2, BEC1286A, and BEEAR2, were also decreased to similar levels as those observed upon inoculation with the *bel2-5* deletion mutant. Moreover, En1282 formed nodules when inoculated with the BEEAR1 and BENLS mutants (Figures 1B,C). These results demonstrate that some domains/motifs (RD1, RD2, C1286A, and EAR2) play pivotal roles in Bel2-5 during nodulation of the *nfr1* mutant soybean, while EAR1 and NLS are less important.

BARC-2 plants inoculated with *B. elkanii* USDA61 formed only a few nodules, whereas those inoculated with the *bel2-5* deletion mutant formed numerous nodules (Figures 1B,C). The nodule numbers derived by BEC1286A also increased to similar levels as those observed for BERD2 and the *bel2-5* deletion mutant, followed by those observed for BERD1, BENLS, and BEEAR2, while plants inoculated with BEEAR1 formed few nodules, similar to those inoculated with WT USDA61 (Figures 1B,C). Together, these results suggest that each domain/motif plays a vital role in the nodulation phenotypes of both En1282 and BARC-2, except for EAR1, which exhibited less importance than the other domains/motifs.

### Differential Expression of Soybean Symbiosis and Defense-Related Genes After Infection With *bel2-5* Mutants

A previous study revealed that Bel2-5 regulates the expression of symbiosis- and defense-related genes in soybean (Ratu et al., 2021). Additionally, in the incompatible interaction between BARC-2 (*Rj4/Rj4*) and *B. elkanii*, the T3SS of *B. elkanii* induced the expression of the soybean defense-related genes *PR-1* and *PDF1.2*, which are known to participate in salicylic acid (SA) and jasmonic acid (JA) signaling to restrict rhizobial infection (Yasuda et al., 2016). To further investigate the importance of each domain/motif of Bel2-5 in the expression of these soybean genes, we compared their expression in the roots of En1282 and BARC-2 inoculated with WT USDA61 and the *bel2-5* deletion mutant and its derivatives using quantitative RT-PCR.

**FIGURE 2:**
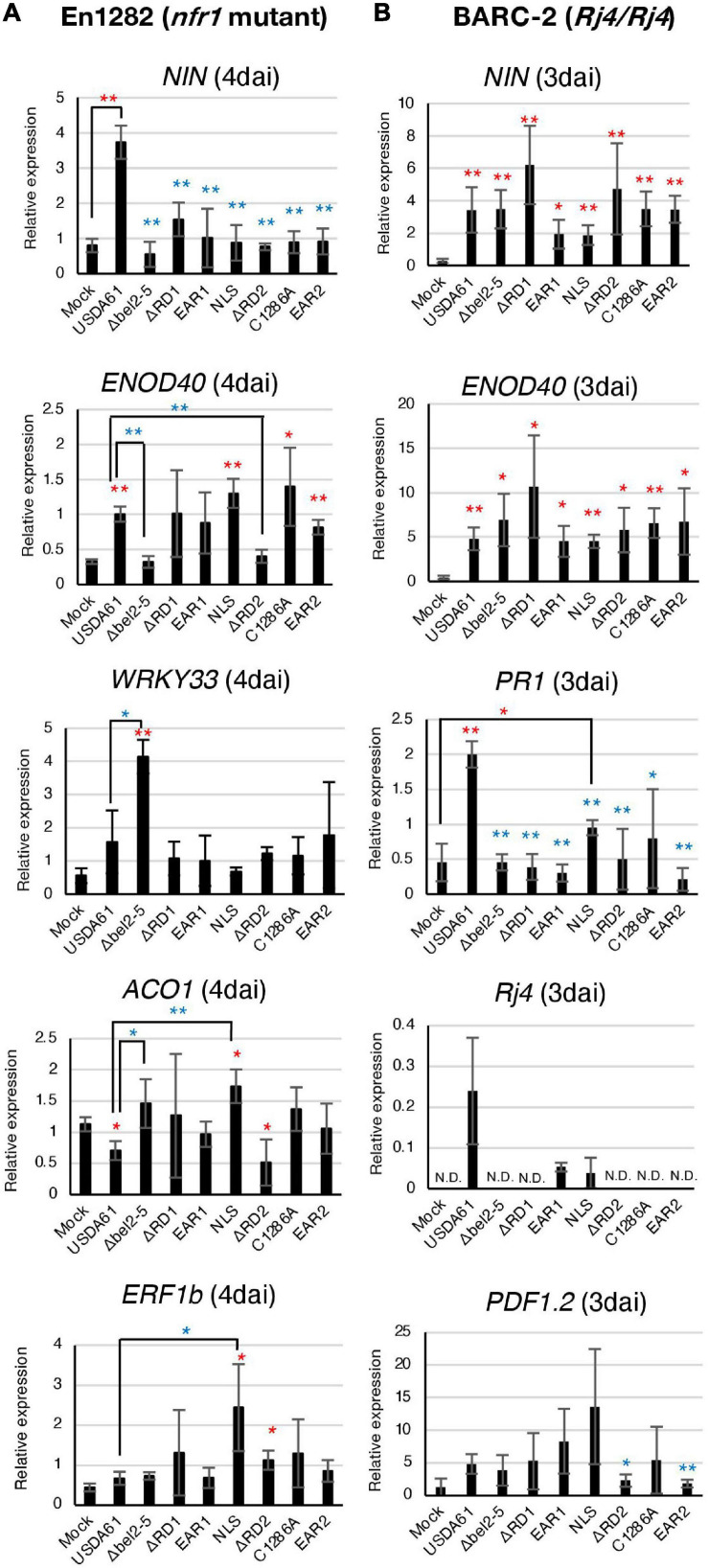
qRT-PCR analysis of soybean defence- and symbiosis-related genes in roots of En1282 **(A)** and BARC-2 **(B)** inoculated with the *B. elkanii* USDA61 wild-type and the *bel2-5* deletion mutant and its derivatives (substitutions or deletions). RNA was isolated from the roots of En1282 and BARC-2 at 4 and 3 days after inoculation (dai), respectively. The expression level of each gene was normalized to that of the *SUBI2-2* gene. The values represent the mean ± SD (*n* = 3–4 independent soybean roots) **(A,B)**. Statistical analyses (Student’s *t*-test) were performed for comparison of each inoculation with the mock control (water) (red asterisk) or wild-type USDA61 (blue asterisk). *P < 0.05; **P < 0.01 **(A,B)**. USDA61, *Bradyrhizobium elkanii* wild-type; Δ*bel2-5*, *bel2-5* knockout mutant; ΔRD1, repeat domain I deletion mutant; BERD1; EAR1, amino acid substitution mutant of the EAR1; BEEAR1; NLS, amino acid substitution mutant of the NLS; BENLS; ΔRD2, repeat domain 2 deletion mutant; BERD2; C1286A, amino acid substitution mutant of the ULP domain; BEC1286A; and EAR2, amino acid substitution mutant of the EAR2; BEEAR2.

In the roots of En1282, the relative expression levels of *NIN* and *ENOD40*, two well-characterized symbiosis genes, were significantly increased by inoculation with USDA61 (Figure 2A). The induction of *NIN* was diminished in the roots inoculated with the *bel2-5* deletion mutant and other mutants. Moreover, the induction of *ENOD40* was diminished only by inoculation with the *bel2-5* deletion mutant and BERD2 (repeat domain 2 deletion mutant) compared with USDA61. Upon inoculation with the other mutants, the expression of *ENOD40* was similar to that upon inoculation with USDA61 and was higher than that in the mock treatment (Figure 2A).

The defense-related genes *WRKY33*, *ACO1*, and *ERF1b* were previously reported to be suppressed by the Bel2-5 effector in the roots of En1282 (Ratu et al., 2021). Suppression was observed for the expression of *WRKY33* and *ACO1*, as inoculation with the *bel2-5* deletion mutant significantly increased the expression of these genes. The suppression of *WRKY33* was not diminished by mutation of each domain/motif. Moreover, for *ACO1* and *ERF1b*, only mutations in the NLS motif significantly increased the expression of these genes compared with that observed with USDA61 (Figure 2A), suggesting that the nuclear localization of Bel2-5 is critical for the suppression of these defense-related genes.

In BARC-2 roots, the expression of the symbiosis-related genes *NIN* and *ENOD40* was significantly induced by inoculation with all the tested strains (Figure 2B). Notably, USDA61 and the BEEAR1 mutant, which formed few nodules on BARC-2 plants, also induced the expression of genes, suggesting that these strains were able to trigger NF signaling but failed to induce nodulation at the later stage. The expression of the *PR1* and *Rj4* genes that were reported to be induced by the *B. elkanii* T3SS (Yasuda et al., 2016) was diminished by mutation of each domain/motif of *bel2-5* to the same level as that observed with the *bel2-5* deletion, while the USDA61 strain triggered their expression (Figure 2B). These results suggest that each domain/motif of the Bel2-5 effector plays pivotal roles in the induction of the expression of the *PR1* and *Rj4* genes.

### Expression of Bel2-5 in Yeast Causes Growth Inhibition

**FIGURE 3:**
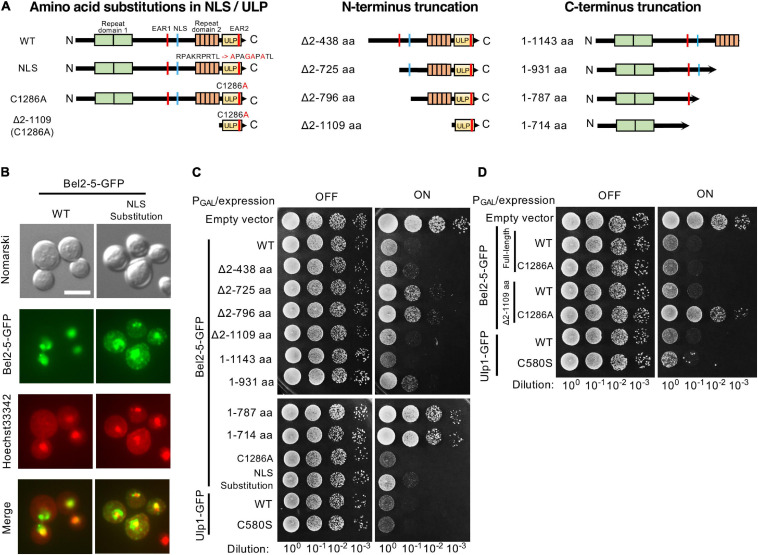
Expression of Bel2-5 and its derivatives in yeast cells. **(A)** Schematic representation of Bel2-5 and its derivatives used for heterologous expression in yeast cells. Amino acid substitutions in the nuclear localization signal (NLS) and catalytic core of the ubiquitin-like protease (ULP) domain of Bel2-5. The Bel2-5 N- or C-terminal truncated constructs. **(B)** Subcellular localization of wild-type Bel2-5 and its mutant on the predicted NLS motif in yeast cells. *Bel2-5* was tagged with a GFP reporter. Yeast cells were stained with Hoechst dye solution as a counterstain for Bel2-5 localization. From top to bottom: unstained yeast cells (Nomarski), the GFP-labeled Bel2-5 effector, yeast cells stained with Hoechst 3342 dye, and combined GFP-tagged Bel2-5 and Hoechst 3342 dye staining. Scale bars: 5 μm. **(C, D)** Expression of the GFP-tagged Bel2-5 effector and its derivative mutants in yeast cells. Bel2-5 expression was under the control of the inducible promoter *GAL1*. The GFP-tagged wild-type yeast Ulp1 and its catalytic core mutant, with a cysteine to serine (C580S) mutation, was used as a control to compare the activity of Bel2-5 in yeast cells.

Effector proteins have been observed to confer a growth inhibition phenotype when heterologously expressed in the yeast *Saccharomyces cerevisia*e (Siggers and Lesser, 2008; Popa et al., 2016). This growth inhibition is thought to be the consequence of the effector-induced compromise of cellular processes conserved between yeast and higher eukaryotes. To investigate the function of the Bel2-5 effector, we expressed Bel2-5 in yeast cells under the control of the *GAL1* promoter and evaluated its effect on yeast growth. As shown in Figure 3C, the expression of WT Bel2-5 caused yeast growth inhibition at a level similar to that of Ulp1, which is a yeast deSUMOylation enzyme. We confirmed that GFP-tagging at the C-terminus of Bel2-5 did not affect its yeast growth inhibitory activity (Supplementary Figure S1). To clarify the determinant domain/motif for the yeast growth inhibitory activity of Bel2-5, a set of mutant derivatives of *bel2-5* was created and analyzed in yeast (Figure 3A). Bel2-5 was localized in the nucleus in yeast, as confirmed by co-staining the nucleus with Hoechst 33342 (Figure 3B, left panels: WT). We confirmed that this nuclear localization was partly dependent on the NLS sequence of Bel2-5 (Figure 3B) and appeared to be partly responsible for the yeast growth inhibitory activity (Figure 3C, lower panel).

Next, we analyzed the N- or C-terminus truncation mutants of *bel2-5* in yeast. The N-terminus truncation mutants Δ2-725 and Δ2-796 exhibited reduced yeast growth inhibitory activity compared with WT, whereas the Δ2-1109 mutant, with the ULP domain only, exhibited yeast growth inhibitory activity similar to that of WT (Figure 3C). Interestingly, the C-terminal truncation mutant, the 1-1143 mutant, which lacked the ULP domain, exhibited even stronger yeast growth inhibitory activity than WT, and the 1-931 mutant showed the same level of growth inhibitory activity as WT (Figure 3C). Furthermore, the C-terminal truncation mutants 1-787 and 1-714, which lacked NLS sequences, lost their yeast growth inhibitory activity, indicating that nuclear localization is important for the yeast growth inhibitory activity of the N-terminal domain.

We further focused on the ULP domain of Bel2-5 and examined the growth of the yeast C1286A mutants, harboring a substitution of a cysteine residue by an alanine residue in the putative active site of the ULP domain (Figures 3A,D). Yeast growth inhibitory activity is still conferred from the full-length Bel2-5 carrying C1286A mutation, while Δ2-1109 truncation with the C1286A mutation exhibited complete loss of the growth inhibitory activity (Figure 3D), indicating that the yeast growth inhibitory activity of Bel2-5 is partly dependent on ULP activity.

Together, these results confirm that Bel2-5 possesses independent functional activities in the N-terminal domain containing the NLS sequence and the C-terminal domain containing the ULP domain.

### Domain Swapping Between *B. elkanii* Bel2-5 and *Xcv*. Effector XopD

**FIGURE 4:**
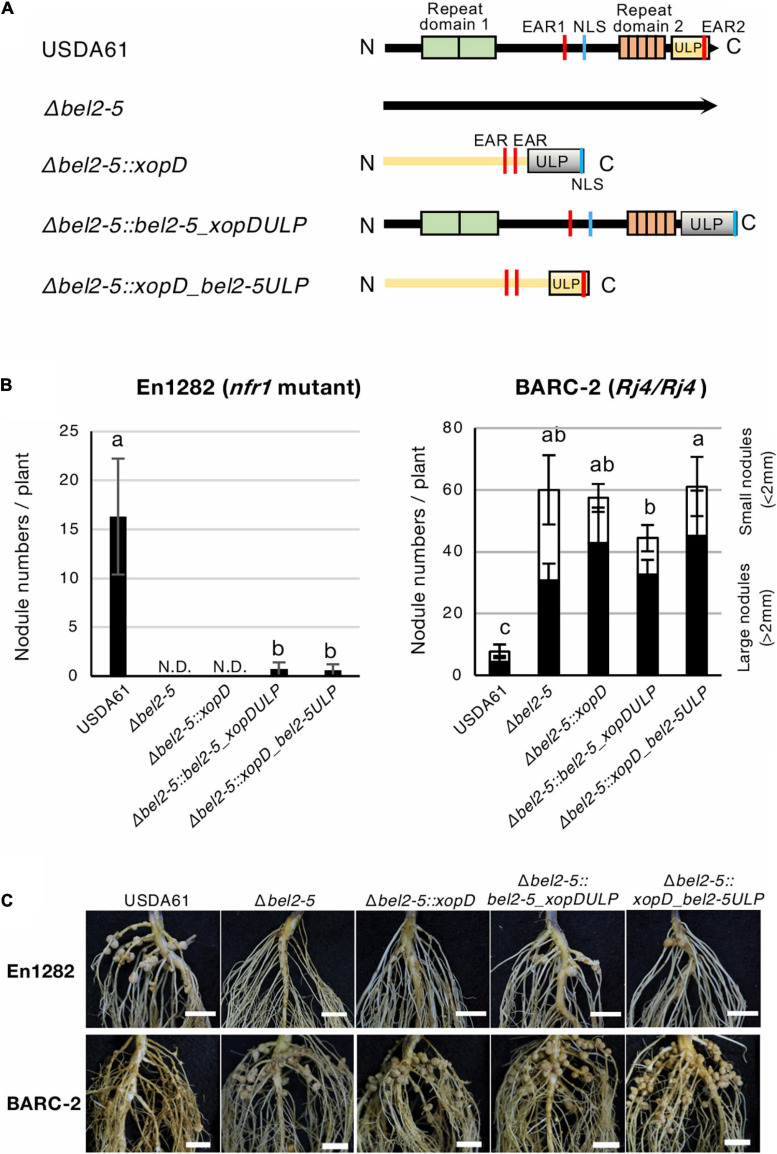
Nodulation phenotypes of *B. elkanii* USDA61 wild-type and the *bel2-5* deletion mutant and its derivatives carrying swapped regions of the ULP-like domain on soybean En1282 (*nfr1* mutant) and BARC-2 (*Rj4*/*Rj4*). **(A)** Schematic representation of swapping of the ULP-like domain between *B. elkanii* Bel2-5 and *Xcv.* XopD. **(B)** Nodulation of *G. max* cv. En1282 and BARC-2 inoculated with wild-type and mutant strains of *B. elkanii* USDA61. Nodule numbers were counted at 30 days after inoculation (dai). The values represent the mean ± SD (*n* = 10 plants, from two independent inoculation tests). N.D., not detected. Means followed by different letters are significantly different at the 0.05 level by Tukey’s test. **(C)** Roots of En1282 and BARC-2 inoculated with wild-type and mutant strains of *B. elkanii* USDA61. Roots were photographed at 30 dai. Scale bars, 1 cm. USDA61, *Bradyrhizobium elkanii* wild-type; Δ*bel2-5*, *bel2-5* knockout mutant; Δ*bel2-5:xopD*, *bel2-5* knockout mutant complemented with *xopD*; Δ*bel2-5:bel2-5_xopDULP*, *bel2-5* knockout mutant complemented with *bel2-5* harboring the ULP domain of *xopD*; Δ*bel2-5:xopD_bel2-5ULP*, *bel2-5* knockout mutant complemented with *xopD* harboring the ULP domain of *bel2-5*.

Both *B. elkanii* Bel2-5 and *Xcv.* XopD carry EAR, NLS, and ULP domains/motifs, and each of them plays an important role in symbiosis and pathogenicity in the interaction with the respective hosts (Figures 1–3, Hotson et al., 2003; Kim et al., 2008, 2013). To clarify whether Bel2-5 and XopD possess unique features to perform their symbiotic/pathogenic functions in the respective hosts or can function as interchangeable effectors, we performed domain swapping between Bel2-5 and XopD (Figure 4A and Supplementary Data S2). Domain-swapped chimaeras were constructed and introduced into the *bel2-5* deletion mutant strain, which was then used for nodulation assays on En1282 and BARC-2 soybean plants. In En1282, the full-length *Xcv.* XopD (Δ*bel2-5:xopD*) did not complement the nodulation capability of the *bel2-5* deletion mutant. Intriguingly, when the ULP domain of XopD was replaced with that of Bel2-5 (Δ*bel2-5:xopD_bel2-5ULP*), nodule formation was observed, although the number of nodules was very small (Figures 4B,C). Moreover, when the ULP domain of Bel2-5 was replaced with that of XopD (Δ*bel2-5:bel2-5_xopDULP*), the nodule numbers were drastically decreased compared with those of WT Bel2-5 (USDA61) (Figures 4B,C).

On the other hand, no significant effect was observed among the nodule numbers of the domain-swapped construct on BARC-2 soybean (Figures 4B,C). These results suggest that the ULP domains of these effectors are partially exchangeable and function similarly during nodulation events, while other parts of Bel2-5 also contain unique features that are involved in the differentiation of species-specific functions in the respective host plants.

## Discussion

In this study, we analyzed the functional domains/motifs of the *B. elkanii* Bel2-5 effector in controlling NF-independent symbiosis with En1282 and incompatible symbiosis with BARC-2 (*Rj4/Rj4*). Bel2-5 possesses two repeat sequences (RD1 and RD2), two EAR motifs (EAR1 and 2), an NLS, and a ULP domain (Ratu et al., 2021, Figure 1). By mutational analysis, we revealed that the Bel2-5-dependent nodulation phenotype requires most of the domains/motifs, as the mutation of any of these domains/motifs caused significant changes in the nodulation phenotype, similar to the *bel2-5* deletion mutant (Figure 1). Notably, the functionality of Bel2-5 was mostly correlated with the growth inhibition effect of Bel2-5 expressed in yeast cells; yeast carrying the Bel2-5 WT sequence and mostly, truncated mutants of *bel2-5* exhibited significant growth inhibition (Figure 3).

Among the domains, the EAR1 motif seems to be less important than the others, as the strain carrying a mutation in EAR1 still induced nodulation on En1282 and exhibited incompatibility with BARC-2 plants (Figure 1). The differential impact of each domain was observed only for the EAR2 motif; the BEEAR2 mutant exhibited complete loss of nodulation capability on En1282, while the inhibition of nodulation in BARC-2 was still present, albeit weakened (Figure 1). This may suggest that Bel2-5 interacts with different host targets in En1282 and BARC-2 via EAR2, leading to differential effects in each host. Except for EAR2, most of the domains/motifs exhibited similar levels of impact on both NF-dependent nodulation in En1282 and nodulation restriction in BARC-2 (Figure 1). This may also suggest that in both En1282 and BARC-2 plants, Bel2-5 targets nucleus via NLS, then binds to the same host target proteins (via EAR1, RD1, RD2) and ULP domain mediates the interaction with host target via deSUMOylation activity. In compatible hosts, this modification of the host target may lead to activation of symbiosis signals, including *NIN* and *ENOD40* genes, ultimately leading to nodule formation. On the other hand, in incompatible hosts such as BARC-2, modification of the host target may be recognized by the host surveillance system and activate ETI, which halts rhizobial infection and nodule development. Identification of host targets that interact with Bel2-5 and are deSUMOylated by Bel2-5 would provide clues to elucidate the molecular mechanism underlying the Bel2-5-dependent nodulation phenotypes.

The expression of Bel2-5 in yeast cells revealed that Bel2-5 possesses independent functional activities in the N-terminal domain containing the NLS sequence and the C-terminal domain containing the ULP domain. Yeast growth was significantly inhibited when the N-terminal domain containing the NLS sequence was expressed (aa 1-1143 and aa 1-931) but not when the NLS was absent (aa 1-787 and aa 1-714) (Figure 3). These results suggest that the N-terminal domain modulates cellular function only in the nucleus, probably by binding with nuclear proteins via repeat domain 1 or the EAR1 motif. Moreover, the C-terminal domain containing the ULP domain (*Δ*2-1109) exhibited inhibitory activity only when the ULP catalytic domain was intact. In yeast cells, Ulp1 is involved in the SUMO modification pathway and has dual functions, processing the SUMO precursor to mature SUMO and deconjugating SUMO from its substrate protein (Li and Hochstrasser, 1999). Similar to Bel2-5, yeast Ulp1 contains an NLS motif on its NH_2_-terminus, which is necessary and sufficient to concentrate the enzyme into nuclear bodies (Li and Hochstrasser, 2003). Interestingly, a previous study on the function of yeast Ulp1 in the cell cycle showed that its activity was separated from nucleocytoplasmic transport; only the catalytic domain of yeast Ulp1 was sufficient to recognize certain substrates and cleave Smt3 conjugates, although localization into the nuclear pore could increase its ability to process SUMO conjugates efficiently (Li and Hochstrasser, 2003). Intriguingly, when only the non-catalytic domain of yeast Ulp1 (without a protease region) was expressed at high levels, it also caused growth impairment due to excessive accumulation of SUMOylated proteins inside yeast cells (Li and Hochstrasser, 2003). Accordingly, we speculate that the ULP domain of Bel2-5 (*Δ*2-1109) may act similarly to yeast Ulp1 and modulate the cell cycle progression of yeast, not only in the nucleus but also in the cytoplasm, resulting in yeast growth inhibition.

Subcellular localization of Bel2-5 in yeast cells showed that disruption of the NLS caused nucleocytoplasmic distribution, but Bel2-5 was still partially concentrated in the nucleus (Figure 3B). This passive diffusion may contribute to partial changes in the nodulation phenotypes of the mutant (Figure 1). Expression analysis of soybean genes showed that suppression of *ACO1* and *ERF1b* in the roots of the *nfr1* mutant required a functional NLS, while *NIN* was expressed at a significantly lower level when the NLS was mutated (Figure 2A). In BARC-2, the expression of at least the *PR1* and *Rj4* genes was induced by infection with the BENLS mutant strain but not by the other mutants (Figure 2B). To date, some rhizobial T3Es have been shown to localize to the nucleus, including NopL of *Sinorhizobium* sp. NGR234 (Ge et al., 2016), ErnA of *Bradyrhizobium* sp. ORS3257 (Teulet et al., 2019), and NopD of *Bradyrhizobium* sp. XS1150 (Xiang et al., 2020). However, whether their cellular/symbiotic activities depend on nuclear localization remains unknown. Furthermore, the role of the NLS sequence in some pathogenic effectors has been demonstrated. For example, the avirulence activity of the *Xcv.* AvrBs3 effector in plant cells was confirmed to be dependent on an active NLS (Van den Ackerveken et al., 1996). Similarly, *Xcv.* HpaA-mediated disease development requires the two functional NLSs of this protein (Huguet et al., 1998). Non-canonical nuclear signals of the gall-forming HsvG, a T3E of the phytopathogen *Pantoea agglomerans* pv. *gypsophilae*, could bind with importin-α proteins of *Arabidopsis*, and NLS deletion abolished the pathogenicity of HsvG (Weinthal et al., 2011). Overall, it is likely that the nuclear localization of Bel2-5 via its NLS motif is critical for Bel2-5-dependent soybean gene regulation and alters nodulation efficiency.

A homology search revealed that Bel2-5 shares high similarity with at least three putative T3 effectors, namely, Blr1693, Blr1705, and Bll8244, of *Bradyrhizobium diazoefficiens* USDA110 (Ratu et al., 2021). Intriguingly, unlike USDA61, USDA110 is unable to form nodules in En1282 and nodulates well in *Rj4* soybean (Okazaki et al., 2013; Hayashi et al., 2014). Structural analysis indicated that these homologs lack repeat domain 1 (RD1, aa 148-438) and the EAR1 motif (aa 715-719) within their sequences. Similarly, repeat domain 2 (RD2, aa 932-1109) of Bel2-5 exists among USDA110 homologs; however, it has low sequence conservation and differs in the number of repetitions (Supplementary Data S3, Ratu et al., 2021). The function of repeat domains in rhizobia remains unknown; however, our phenotypic test showed that mutations in repeat domains of Bel2-5 significantly affected the nodulation phenotype (Figure 1). The pathogenic T3 effector HsvG possesses 71-aa and 75-aa tandem repeats that function as transcriptional activators and are involved in host specificity (Nissan et al., 2006). The T3 effector transcriptional activator-like (TAL) of *Xanthomonas* spp. carries a unique tandem 34-aa repeat domain that determines DNA binding specificity. DNA-binding effector proteins induce the expression of host susceptibility genes to promote *Xanthomonas* colonization (Richter et al., 2014). The repeat sequence was also found in the *Ralstonia solanacearum* T3 effector RipTAL1, which activates transcription of host susceptibility genes (de Lange et al., 2013). Furthermore, it has been reported that diverse variations in the sequence composition and number of protein domain repeats allow flexible binding to multiple binding partners (Björklund et al., 2006; Boch et al., 2009). Together, the repeat domains of Bel2-5 and USDA110 effector homologs may be involved in the recognition of specific host proteins, resulting in different symbiotic phenotypes.

Of particular interest is the species-specific function of the *B. elkanii* Bel2-5 and *Xcv.* XopD effectors. Bel2-5 shares a high degree of homology with XopD, and both effectors contain ULP domain with a catalytic triad (H, D, and C), which is well conserved (Ratu et al., 2021). A nodulation test using ULP domain-swapped *bel2-5* and *xopD* mutants revealed that both Bel2-5 carrying ULP of XopD and XopD carrying ULP of Bel2-5 were capable of inducing the formation of only a limited number of nodules on En1282 (Figure 4). These results indicated that both the ULP domain and upstream region of the Bel2-5 effector are required for triggering NF-independent nodulation signaling in soybean cells. Similarly, in BARC-2 soybean, domain-swapped mutants induced the formation of numerous nodules, similar to the effect of *Δbel2-5*, indicating that both the ULP domain and upstream region of Bel2-5 are required for the induction of ETI-like responses leading to nodulation restriction. These results may imply that both the ULP domain and upstream region of Bel2-5 work together, e.g., the upstream region recruits and interacts with the host target, and the ULP domain deSUMOylates it to trigger signaling, leading to Bel2-5-dependent nodulation. In addition to the conserved ULP domain, Bel2-5 and XopD also share some features, including EAR motifs and their ability to accumulate in the plant nucleus via the NLS motif. Thus, other parts, such as the DNA-binding domain (DBD) in XopD and repeat domains in Bel2-5, are likely involved in the interaction with their respective target in host cells and are thus responsible for species specificity.

A previous study reported that the *Xcv.* strain colonizes tomato plants by suppressing ET production via XopD-mediated deSUMOylation of the SIERF4 transcription factor (Kim et al., 2013). Structural and functional study of the XopD effector revealed that the DBD and EAR motifs were involved in the critical step for the substrate specificity or enzyme kinetics of its SUMO protease activity (Kim et al., 2013). Intriguingly, our present study revealed that the EAR motifs in Bel2-5 were essential for its symbiotic functions, with EAR1 playing a less important role than EAR2 (Figure 1). When expressed in yeast cells, the EAR1-deleted mutant (Δ2-725 aa) showed weaker inhibitory activity than that carrying EAR1 (Δ2-438 aa) (Figure 3). To our knowledge, Bel2-5 is the only rhizobial effector reported to have functional EARs that determine the interaction with host plants. The EAR motifs were found to be highly conserved, at least within Bel2-5 rhizobial homologs (Ratu et al., 2021). Notably, the pattern of EARs in Bel2-5 was different from that in *Xcv.* XopD but similar to that in PopP2, a T3 effector of *R. solanacearum* that causes bacterial wilt in a wide range of host plants (Genin and Denny, 2012). In plant cells, PopP2 is recognized by the RPS4/RRS1 protein complex via direct binding with the WRKY domain on the RRS1 C-terminus, resulting in the activation of defense responses (Le Roux et al., 2015; Sarris et al., 2015). Mutation in the EAR motif of PopP2 impaired not only the avirulence activity of *R. solanacearum* but also bacterial virulence and stability in plant cells (Segonzac et al., 2017). The expression of marker genes, such as *PR1*, *FMO1*, *PBS3*, and *SARD1*, was induced by T3S-derived PopP2, but their expression was diminished by the *R. solanacearum* EAR-mutated strain (Segonzac et al., 2017). Notably, we also found that Bel2-5 EAR motifs determine the transcriptional level of soybean defense genes, including *WRKY33*, *PR1*, *Rj4*, and *PDF1.2* (Figure 2). How these EAR motifs modulate the expression of soybean genes and control nodulation remains unclear. As EAR motifs are known to facilitate protein-protein interactions in transcriptional complexes and function in transcriptional repression (Kazan, 2006), it is likely that the Bel2-5 effector interacts with plant transcription complexes via EAR motifs and controls host stress and defense responses in the nuclear membrane, as reported in pathogenic effectors (Kim et al., 2008; Segonzac et al., 2017).

In conclusion, we identified the functional domains/motifs of Bel2-5 responsible for NF-independent symbiosis in the *nfr* soybean mutant and nodulation restriction in *Rj4* soybean. Bel2-5-dependent symbiosis requires most of the predicted domains/motifs, as the mutation of each domain/motif significantly affected the symbiotic phenotypes, as well as the expression levels of symbiosis- and defense-related genes in soybean roots. Nodulation phenotypes of the domain-swapped mutants of *bel2-5* and *xopD* indicated that both the ULP domain and upstream region are required for Bel2-5-dependent symbiotic phenotypes (Figure 4). While XopD suppresses the defensive response (Kim et al., 2008), Bel2-5 has the dual function of triggering the symbiotic signal and suppressing the defensive response, suggesting that Bel2-5 has evolved to perform a symbiotic function during coevolution with host legumes. Identification of the Bel2-5 host target and its modification by the effector would provide clues to elucidate the mechanism of Bel2-5-dependent symbiotic signaling and shed light on the evolution of this effector from pathogenicity to symbiotic utilization.

## Data Availability Statement

The original contributions presented in the study are included in the article/Supplementary Material, further inquiries can be directed to the corresponding author.

## Author Contributions

SR, AH, MT, and SO designed the research. SR, AH, and CK performed the research and analyzed data. MY contributed new reagents and analytic tools. SR, MT, and SO wrote the manuscript. All authors contributed to the article and approved the submitted version.

## Conflict of Interest

The authors declare that the research was conducted in the absence of any commercial or financial relationships that could be construed as a potential conflict of interest.

## Abbreviations

NFs: Nod factors
T3SS: type III secretion system
T3Es: type III-secreted effectors
MAPK: mitogen-activated protein kinase
ETI: effector triggered-immunity
RD: repeat domain
EAR: ethylene-responsive element-binding-factor-associated amphiphilic repression
NLS: nuclear localization signal
ULP: ubiquitin-like protease
SA: salicylic acid
JA: jasmonic acid
*NIN*: nodule inception
*ENOD40*: early nodulin 40
*ACO1*: 1-amino-cyclopropane-1-carboxylate (ACC) oxidase
*ERF1b*: ethylene response factor 1b
*PR1*: pathogenesis-related protein 1
dai: days after inoculation
USDA61: *Bradyrhizobium elkanii* wild-type
Δ *bel2-5*: *bel2-5* knockout mutant
Δ RD1: repeat domain I deletion mutant
BERD1; EAR1: amino acid substitution mutant of the EAR1
BEEAR1; NLS: amino acid substitution mutant of the NLS
BENLS, Δ RD2: repeat domain 2 deletion mutant
BERD2, C1286A: amino acid substitution mutant of the ULP domain
BEC1286A and EAR2: amino acid substitution mutant of the EAR2
BEEAR2, Δ *bel2-5:xopD*: *bel2-5* knockout mutant complemented with *xopD*
Δ *bel2-5:bel2-5_xopDULP*: *bel2-5* knockout mutant complemented with *bel2-5* harboring the ULP domain of *xopD*
Δ *bel2-5:xopD_bel2-5ULP*: *bel2-5* knockout mutant complemented with *xopD* harboring the ULP domain of *bel2-5*
