# Supplementary material for: Multiple Domains in the Rhizobial Type III Effector Bel2-5 Determine Symbiotic Efficiency With Soybean
Source: Front Plant Sci. 2021 Jun 7;12:689064. doi: 10.3389/fpls.2021.689064 (PMC8215712; doi:10.3389/fpls.2021.689064)
Supplement: Supplementary file 1 [file Data_Sheet_1.docx]

***Supplementary Material***

**Multiple Domains in the Rhizobial Type III Effector Bel2-5 Determine Symbiotic Efficiency with Soybean**

**Safirah Tasa Nerves Ratu^1^, Atsushi Hirata^2^, Christian Oliver Kalaw^3^, Michiko Yasuda^3^, Mitsuaki Tabuchi^2^, and Shin Okazaki^1,3^**^*^

*** Correspondence:** Shin Okazaki:[sokazaki@cc.tuat.ac.jp](mailto:sokazaki@cc.tuat.ac.jp)

**This file contains:**

**Supplementary Data S1.** DNA fragment containing *bel2-5* and its promoter region

**Supplementary Data S2.** Swapping region of ULP-like domain between *B. elkanii* Bel2-5 and *Xcv.* XopD effectors

**Supplementary Data S3.** Alignment of Bel2-5 effector and its homologs in *B. japonicum*

USDA110

**Supplementary Figure S1.** GFP-tagging at the C-terminus of Bel2-5 did not affect its yeast growth inhibitory activity

**Supplementary Table S1.** Soybean seeds, bacterial or yeast strains, oligonucleotides, and plasmids used in this study

**1 Supplementary Data**

**Supplementary Data S1.** DNA fragment containing *bel2-5* and its promoter region. The sequence was used as a template for generating *bel2-5* derivatives (substitution or deletion). The sequence consists of: (1) *bel2-5* coding sequence (3987 bp); (2) upstream region of *bel2-5* encoding promoter sequence (510 bp), and (3) downstream region of *bel2-5* (45 bp). The ATG start- and TGA stop-codons are underlined. The promoter sequence of *bel2-5* is boxed.

﻿TTCGTCTATTGGGGTGACGTGGGTGTCATTCTGCAATTCTGCCCCCCTAAGCCGGGGGATCGGCGTCCAAAATTTGCAAGCCGATTCAGCAATAGGCGTCTTAAGATCAATACTGAATTGTAAGGCACCAAAAGTGGTTGCGTTTCGATTTTTGCCTCGCGCGTTCAGCGCATCCTGTCAAGATGTTGGCTGGAGCCGCGAAAGCTGGCCGTACGCCTCACCGATGAGAAGCAGTATTTTGTCTCTGAATCTTCGGTATCTTCGGTCTCTCGGCTGTTGGCGGCGAAGCGCGCAGGCACATGCACCATGCTGTCTTGGCGCGGCAATTTTCGTCATCAGGTTCTCGACAGCTTCCCCATTTAACAAAACATACAGGGCTGGTCGCCTTGCAAAAACATCGGCTGCCGATGCTCACGGTGCGCGCCTGTCGAGGGCCGTAAAGGGTGGTTTGTTGCTGATTCCCAGCATGTCAGATCGAAGCCCATGTTCATATTTTGAAGGTGAGAACGG**ATGGATTTCCCCTCGACCAAGTCGGTGCATGAACAATCGGACAGCACGGGCCCGCAAGAGAGCTCACCCGCGGCTCCGTCCGCAGCCGCGGCGACCTTTGAGCGGCAATTGAGCGAGATCGCCAACTCAGATGGAGGTGGTGGAGCAATGCCGGCCGCCTCGGCGCTGCAGTCGGCTCAGTCAACTTGGGTTTTGATAGGACGGCAGGGCAAGCATCTCCTTTATTCCGAGGACGCTCGCCTTATTTCGGGGCTTGAGAAGGCCCTTATCGGCAACCCCACCCACCGCAACGCTGGGGACTATCTAACTTCACTTCGCAGCTTTGGCGGCTGGCTCTTCGCAAATAACAAAACGAGCATTGTTGCTCGGCTCAACGACCCGTCGCTGACCCATGATGCACGCGAGTTCGAAAAGAGGCGTCCCTCGAATATCCTTGCGGCATTGAACCATCTCCGGACCTTCCAGTCGACGGGCGGAGTGACGGTCACAGCCCGCACTGAGCTAAATCCTCACCCTCAGGACGCGGATCTCATCAACGAGTACAAAAAAGAAACAGCGACAAGTACCGGCAGGATGTATGCAACTGCTCTTCGCAGTTTCGGTCACTACCTGCGTGAAAACAACAAGAAGGGCATTGCTACTCGGCTTTCCGGCGGGGCGTTGGATGAAGATGTCAATAGCTATAAGAAGGGCGCCGGTGCTGATTCGAGGATCGGTGCCGCTCTGGCTCAACTCCGAAAATCGCAGGCCGGCGCTAAGGCGATGGAGCCCGAGCGCCATTTTGATCCCAAAGACGCGGCCCTGATGGAGTCGATGCAGGTCGGCGACGCCGCTGCGCAGCACAGTGCGTCGCAGCAAGCTGGCAGTTGGTCAGAGGAATTGAACCATCTCCGGACCTTCCAGTCGACGGGCGGAGTGACGGTCACAGCCCGCACTGAGCTAAATCCTCACCCTCAGGACGCGGATCTCATCAACGAGTACAAAAAAGAAACAGCGACAAGTACCGGCCGGATGTATGCAACTGCTCTTCGCAGTTTCGGTCACTACCTGCGTGAAAATAACAAGAAGGGCATTGCTACTCGGCTTTCCGGCGGGGCGTTGGATGAAGATATCAATAGCTATAAGAAGGGCGCCGGTGCTGATTCGAGGATCGGTGCCGCTCTGGCTCAACTCCGAAAATCGCAGGCCGGCGCTAACGCGATGGAGCTCGAGCGGCATATTGATCCCAAAGACGCGGCCCTGATGGAGTCGATGCAGGTCGGCGACGCCGCTGCGCAGCACAGTGCGTCGCAGCAAGCTGGCAGTTGGCCAGAGGAATTTCTTCCTGCGGAAGGCCACGATCAGGATTTGGGCCGGATGGACGAACCCGGCCCGTCGTCGTCGGCGCCGCAGCCGGCTCAGTCAACTGGGATTTTGAGAGGGCGGAGGAAGCCTCTTTATTCCGAGGATGCTCCCCTTATTTCGGGGCTTGAGGAGGCCCTCCGCAGTGGCAACGCCGCCGAACGCACCGCCAAAGACCTTGTAGGCCCGCTTCGCGCCTTTGGCCGCTGGCTCTTGGCAAATAACAAAACGAGCATTGTTGATCGGCTCGAAAAAGAGTCGCTGACCGATGATGCGCGTGAGTTCATCGAAAAGGGTAAGGGCAGTCGCCTCCTTATTAGGTCAATAGGTCTTCTCCGGACCTTTCAGTCGACGGGCGGAGTGACGGTCACAGCCACCACTGAGCTAAATCCTTACCTCCAGGACGCGGCTCTCATCATAGAGTACCAAAACGAAGCAGCGACAAGTACCGGCGGTGTGTATGCGACTGCTCTTCGCAGTTTCGGTCACCACCTGCGTGAAAATAACAAGAAGGGCATTGCTACTCGGCTTTCCGGCGGGGCGTTGGATGAAGATGTCGAGGCCTATAAAAAGGACTTCGGTGGCATCCGGACGATCGATGCCGCATTAGGTCAACTCCGAAAATCACAGGCGGGCGCTAAAGCGATGGAGCGCGAGCGCTATATTTCCCCCGGTCCTGATCCCGAAGGCGCGGCACTGATGGAGCCGAGGCGGGCCGGCGACGCCGCAGCGCAGCACAGCTCGCAGGAAGTTGGCAGTTGGCCAGAGAAACTGCTTCCTGCGGAACGCCACGAGCAGGATTTGGTTTTGGGGCTGATGGACGAACCCGGCCCGTCGTCATCTCTCGAGCCAGTCGCGCGGCACGACCAGGCATCGGATCCCGGAGATTCCATTCGTCCCCTGAACTGGCGCCGGGACGGCCAGCAGTTCTCGGAAGAGCCGATGGCTGCACTTGCCAGGAGCAACCTCCCGCCAAGCGAGGAGATCCTCATCAACGATGAACAGGATGCAGCTGAGTTAAGGCCAGCGAAGAGGCCGAGGACCCTAGACAATCCGCAAGGCCTTGCTATTGAGCGGCTGCTGAGCGAGATCGCCGCGACCCCGGCCCCCACCCATCAACAGGGTGCATCGCCATGGCATGCGCAGCCGATGATGCAGGCGAGCGGGCACGAAGATGCAACGGCGCCGCATGCGGCCGCGACGTACGTCGCGGGCGCCGCCGCGCAGCACAGCGCGCCGCAGGGAGCTGTCAGTCGGCCATTGGTCCTCCCGGAAGGTTACGACCGGGATCTGCGTTTGATGGGGAAAGACGGCCCATCGTGGCCCGAGGTTCCCCCTGGGCAGGCGCAGGACATAGTCCAAGCTGGACGGCAGCAACCTGCGTGGTCCGCCTCAACCTGGTCGCCGCAGATGCCGCTCGACTTTGATTGGAGTATGTGGCCGACGCTGGAAGCAGCGCCGGCGCCGGCTGCCAGGGCTCGCTCAGGCACCTACGGCGGTCTTGAGTCATTGGTGCATCTGGATGCGCCCACGCCGTCCGAATTGCGCGACGATGCTCACTTTGCGCCGGCGCCCGCTGCCAGGGCTCGCTCAGGCACCTACGGCGGTCTTGAGTCATTGGTGCATCTGGATGCGCCCACGCCGTCCGAATTGCGCGACGATGCTCACTTTGCGCCGGCGCCCGCTGCCAGGGCTCGCTCAGACACCTACGGCGGTCTTGAGTCATTGGTGCATCTGGATGCGCCCACGCCGTCCGAATTACGCGACGATGCTCACTTTGCGCCGGCGCCCGCTGCCAGGGCTCGCTCAGACACCTACGGCGGTCTTGAGTCATTGGTGCATCTGGATGCGCCCACGCCGTCCGAGTTGCGCGACGATGCTCACTTTGCGGCGGCGCCTTTTGCCAGGGCTCGCTCAGACGCCTACCGCGGTTTTCCATTGGTCGATCTGACTGCGCCCACGCCGTCCGAATCACGTGACGATGCTAATTCTGTACGCCCGTTTCCGAGCACCTCCGCTAATGCTCAGATCGGGGCTTTAGATCCGACAGTCTCGTCTCACGGCCACGGGCTGGTGCTCGATGACACAGAATGGCTGGGCGACCAGCATATCGACAGGGATTACGGGCTCCAGGAGCAGGATTTGCAGAGGAACGATCCGGATCTCGCCGCCCGGACGCGGTTCGTGAATCCCCTCATCGCCCTAAATTATCTGCGCTCTAACGACGATGGCGTCGTGCTAACCGAGTTCCAGCGCATCGTCTATGACGATAATGGTAATGATACAGCCGACTTCCTGTTCCTGCCCGTGATTAATGGCAATCCTGAAGATCCTAATAGCCGCGGCAACCATTGGTCGCTGCTGTTCGTAGATCGCAGCGACCGGTGGCGGCCGGTCGCCTATCACTACGATTCCTACGGCGGACTCAACAACAGAGATGCAGCACATCTCGCAAGAAGGCTGAACCTCCCCCTGGAGCTAGCCGACATGGCCCAGCAGCAGAACACTTATGATTGCGGCGTCTTTGTCGTGGACGGCACGCGGGAGCTGGTTAGGCAATTGGCGCAAGGATGGGAGCCAGACCAGCTGAACCTTAGCAACGTCGTTGCCAATCGGCAGGCGCTGCAGAACCGACTCAGGGGTTGA**TGTCGCCGTGGGCGGATAGCTCAGGCTGGAGCGGCTCACGCCGAC

**Supplementary Data S2**. Swapping region of ULP-like domain between *B. elkanii* Bel2-5 and *Xcv.* XopD effectors. **(A)** deletion mutant of *bel2-5* complemented with *xopD* (*Δbel2-5::xopD*), **(B)** deletion mutant of *bel2-5* complemented with *xopD* harbouring ULP domain of *bel2-5* (*Δbel2-5::xopD_bel2-5ULP*), and (**C**) deletion mutant of *bel2-5* complemented with *bel2-5* harbouring ULP domain of *xopD* (*Δbel2-5::bel2-5_xopDULP*). Sequences belong to *bel2-5* or *xopD* are marked with blue or brown colors, respectively. Sequences were amplified without stop codon but encoding the 3xFLAG epitope at their 3’-end (underlined and indicated in red). The ATG start codon is underlined. The promoter sequence of *bel2-5* is boxed. The *xopD* accession number of *X. campestris* pv. *vesicatoria* is BK007963.1

1. ***Δbel2-5::xopD***

**﻿﻿**CTAAAGGGAACAAAAGCTGGAGCTCGGGGTGACGTGGGTGTCATTCTGCAATTCTGCCCCCCTAAGCCGGGGGATCGGCGTCCAAAATTTGCAAGCCGATTCAGCAATAGGCGTCTTAAGATCAATACTGAATTGTAAGGCACCAAAAGTGGTTGCGTTTCGATTTTTGCCTCGCGCGTTCAGCGCATCCTGTCAAGATGTTGGCTGGAGCCGCGAAAGCTGGCCGTACGCCTCACCGATGAGAAGCAGTATTTTGTCTCTGAATCTTCGGTATCTTCGGTCTCTCGGCTGTTGGCGGCGAAGCGCGCAGGCACATGCACCATGCTGTCTTGGCGCGGCAATTTTCGTCATCAGGTTCTCGACAGCTTCCCCATTTAACAAAACATACAGGGCTGGTCGCCTTGCAAAAACATCGGCTGCCGATGCTCACGGTGCGCGCCTGTCGAGGGCCGTAAAGGGTGGTTTGTTGCTGATTCCCAGCATGTCAGATCGAAGCCCATGTTCATATTTTGAAGGTGAGAACGG**ATGGACAGGATATTTAATTTCGACTATAAAAAATATCGCGAAATGACGGAAGCGGCGGATGATTACCGCAATTCTCCGCCTCATGAAGAACAACGGGAAAACCACGGCGCGGGTTATAACATGCATCCGTTGCTTGAATCTCTCCCACGAAGAAATCCTACGCAAGTGCATGCCGATGGCTCGGTTCATCAGATGCGTGCGGCCGCGCCGACCTCCAGAACCCATAGGGATTACTTAAAAATATTAGAACTAATAAGCGCCTATGGCGATGGCAAAGGTATTCCCGAGCTACAAAGAAGCTTTCCAAGCTTTGCTGCCTTTTTGATGGACAGTGGCTTGTCGCATGTAAATGGCAGGCAGATGCTTCAGGAACTGAATGAAGATCAACGCGACCAAGTCATACATCAAATAATAAGACGAATTGAGTATTGTGCGGATCCTGAATATCGAGAAGTTGCGCTGAGCCGACTGGAGTCGGATTGCAGTGGAAAAATTACGCTAAGTCAACGGACTTTGGATCGCATTGACAAAGCCAAAGCCAAAGCCGAAGCCGAAGCCGAAGCCAAAGCCAAAGCCAAAGCCGAAGCCAAAGCCGAAGCCAAAGCCAGAGTCGAAGCCGGAGCCCAATGCAAGATCAACGAAATTATGGAATATATACCAAGATATGAAGCATTAGAGAAAGTGCCAGTGCGTGTGAGATTCCATGCTTACCTGCGTGGTGATGGCTCATTCGGCCCAGGGCTACCTGGCATCCTTCGATACATGACCCCAGATCAGAAGAAAAGATTGTATCTAGCAAGTGAGAGACGCAAACTGGCCTTGGCCGCTCCAAAAAGCAAGCCCACTCCAAAAAGCAAGCCTCTAAAAGGCGTATTCCGGACCCTCCATCAAAAACCAAATTTGCTTCTTGAGATTTCGAGCAAATTCAGCAATAGAGCGTACAGCATCAATGATTCAAGCAGCGGATATTTATCACAAGCAGACCTGGAAGAAATGGTCGACGAGGAAACCGGCGAACTGACTCGTCTGGGTGAAGCAGTGATTTCAGGAGCATCCCAAGGCATCCAGACGGCAATTCGAGCCAACTTCAGAATGCGTTATCAACAACCGGATCTGCCTCCATACAGTCCCCCTCAGGCCTTCCATCGGCCAGAAGAAACGTGGAATCCCCATACTCCGGCGGGTTCTTCCTATTCGTCCCTGTTCCCGCCCACCCCTTCTGGCGGTTGGCCGCAGAACGCATCAGGTGAGTGGCATCCCGATACTCCGGCGGGTTATTCCCATCGTGCATGGCCAGCCCAGCCCGAAGCGTCGAGTTCCACCTTCGATGATCTTGAGTCCTTGGACTATAGGCAGAACTATGGTTATCGCGAATTCGACCTTAACACCCCCCAGGAAATCGAGCAGCCAGGGTGGTGGCAGCAAGCCACGCCCGCCCAAAGCACGGACTCGACCTTCGATGGCCTCTCCTCCATGAGCCATTACGGTAGCGAATTCGACCTCAACATCCCCCAGCAAGAAGAGTACCCTAATAACCATGGCACGCAGACCCCCATGGGATATTCGGCCATGACTCCTGAAAGGATCGATGTGGACAATCTGCCGTCGCCCCAGGACGTCGCAGACCCCGAACTTCCTCCAGTGAGGGCCACTTCGTGGCTGCTGGATGGACATTTGCGCGCCTACACCGATGACCTAGCTCGCCGATTGCGAGGGGAGCCCAACGCCCATTTACTCCACTTTGCCGACTCGCAGGTAGTGACCATGCTGAGCTCCGCAGATCCAGACCAACAGGCCCGCGCACAGCGCCTTCTTGCCGGAGACGACATCCCACCTATCGTGTTCCTGCCGATCAATCAGCCCAACGCTCATTGGTCATTGCTCGTCGTCGACCGGCGTAACAAGGACGCTGTTGCGGCCTACCACTATGATTCCATGGCACAGAAGGACCCACAGCAACGCTACCTTGCTGATATGGCGGCCTATCACCTTGGCCTTGATTATCAACAAACTCATGAAATGCCCATCGCGATACAGTCGGACGGTTATTCCTGCGGCGATCATGTGCTGACCGGGATAGAGGTGTTGGCCCACAGGGTACTCGACGGCACCTTCGACTACGCAGGCGGCAGGGACCTGACTGATATCGAACCAGACCGCGGCCTCATCAGGGATCGTCTTGCCCAAGCGGAGCAAGCTCCAGCAGAAAGCAGCATCAGGCAAGTTCCCGCACGATCCAACGAACAGAAGAAAAAGAAAAGCAAGTGGTGGAAAAAGTTC**CCCCGGGGATCCTCTAGAGTCGACCTGCAGGATTACAAGGATGACGACGATAAGGACTATAAGGACGATGATGACAAGGACTACAAAGATGATGACGATAAATAGGCATGCAAGCTTGGCACTGGTACCCAATTCGCCCTAGCCCG

1. ***Δbel2-5::xopD_bel2-5ULP1***

**﻿**CTAAAGGGAACAAAAGCTGGAGCTCGGGGTGACGTGGGTGTCATTCTGCAATTCTGCCCCCCTAAGCCGGGGGATCGGCGTCCAAAATTTGCAAGCCGATTCAGCAATAGGCGTCTTAAGATCAATACTGAATTGTAAGGCACCAAAAGTGGTTGCGTTTCGATTTTTGCCTCGCGCGTTCAGCGCATCCTGTCAAGATGTTGGCTGGAGCCGCGAAAGCTGGCCGTACGCCTCACCGATGAGAAGCAGTATTTTGTCTCTGAATCTTCGGTATCTTCGGTCTCTCGGCTGTTGGCGGCGAAGCGCGCAGGCACATGCACCATGCTGTCTTGGCGCGGCAATTTTCGTCATCAGGTTCTCGACAGCTTCCCCATTTAACAAAACATACAGGGCTGGTCGCCTTGCAAAAACATCGGCTGCCGATGCTCACGGTGCGCGCCTGTCGAGGGCCGTAAAGGGTGGTTTGTTGCTGATTCCCAGCATGTCAGATCGAAGCCCATGTTCATATTTTGAAGGTGAGAACGG**ATGGACAGGATATTTAATTTCGACTATAAAAAATATCGCGAAATGACGGAAGCGGCGGATGATTACCGCAATTCTCCGCCTCATGAAGAACAACGGGAAAACCACGGCGCGGGTTATAACATGCATCCGTTGCTTGAATCTCTCCCACGAAGAAATCCTACGCAAGTGCATGCCGATGGCTCGGTTCATCAGATGCGTGCGGCCGCGCCGACCTCCAGAACCCATAGGGATTACTTAAAAATATTAGAACTAATAAGCGCCTATGGCGATGGCAAAGGTATTCCCGAGCTACAAAGAAGCTTTCCAAGCTTTGCTGCCTTTTTGATGGACAGTGGCTTGTCGCATGTAAATGGCAGGCAGATGCTTCAGGAACTGAATGAAGATCAACGCGACCAAGTCATACATCAAATAATAAGACGAATTGAGTATTGTGCGGATCCTGAATATCGAGAAGTTGCGCTGAGCCGACTGGAGTCGGATTGCAGTGGAAAAATTACGCTAAGTCAACGGACTTTGGATCGCATTGACAAAGCCAAAGCCAAAGCCGAAGCCGAAGCCGAAGCCAAAGCCAAAGCCAAAGCCGAAGCCAAAGCCGAAGCCAAAGCCAGAGTCGAAGCCGGAGCCCAATGCAAGATCAACGAAATTATGGAATATATACCAAGATATGAAGCATTAGAGAAAGTGCCAGTGCGTGTGAGATTCCATGCTTACCTGCGTGGTGATGGCTCATTCGGCCCAGGGCTACCTGGCATCCTTCGATACATGACCCCAGATCAGAAGAAAAGATTGTATCTAGCAAGTGAGAGACGCAAACTGGCCTTGGCCGCTCCAAAAAGCAAGCCCACTCCAAAAAGCAAGCCTCTAAAAGGCGTATTCCGGACCCTCCATCAAAAACCAAATTTGCTTCTTGAGATTTCGAGCAAATTCAGCAATAGAGCGTACAGCATCAATGATTCAAGCAGCGGATATTTATCACAAGCAGACCTGGAAGAAATGGTCGACGAGGAAACCGGCGAACTGACTCGTCTGGGTGAAGCAGTGATTTCAGGAGCATCCCAAGGCATCCAGACGGCAATTCGAGCCAACTTCAGAATGCGTTATCAACAACCGGATCTGCCTCCATACAGTCCCCCTCAGGCCTTCCATCGGCCAGAAGAAACGTGGAATCCCCATACTCCGGCGGGTTCTTCCTATTCGTCCCTGTTCCCGCCCACCCCTTCTGGCGGTTGGCCGCAGAACGCATCAGGTGAGTGGCATCCCGATACTCCGGCGGGTTATTCCCATCGTGCATGGCCAGCCCAGCCCGAAGCGTCGAGTTCCACCTTCGATGATCTTGAGTCCTTGGACTATAGGCAGAACTATGGTTATCGCGAATTCGACCTTAACACCCCCCAGGAAATCGAGCAGCCAGGGTGGTGGCAGCAAGCCACGCCCGCCCAAAGCACGGACTCGACCTTCGATGGCCTCTCCTCCATGAGCCATTACGGTAGCGAATTCGACCTCAACATCCCCCAGCAAGAAGAGTACCCTAATAACCATGGCACGCAGACCCCCATGGGATATTCGGCCATGACTCCTGAAAGGATCGATGTGGACAATCTGCCGTCGCCCCAGGACGTCGCAGACCCCGAACTTCCTCCAGTGAGGGCCACAGAATGGCTGGGCGACCAGCATATCGACAGGGATTACGGGCTCCAGGAGCAGGATTTGCAGAGGAACGATCCGGATCTCGCCGCCCGGACGCGGTTCGTGAATCCCCTCATCGCCCTAAATTATCTGCGCTCTAACGACGATGGCGTCGTGCTAACCGAGTTCCAGCGCATCGTCTATGACGATAATGGTAATGATACAGCCGACTTCCTGTTCCTGCCCGTGATTAATGGCAATCCTGAAGATCCTAATAGCCGCGGCAACCATTGGTCGCTGCTGTTCGTAGATCGCAGCGACCGGTGGCGGCCGGTCGCCTATCACTACGATTCCTACGGCGGACTCAACAACAGAGATGCAGCACATCTCGCAAGAAGGCTGAACCTCCCCCTGGAGCTAGCCGACATGGCCCAGCAGCAGAACACTTATGATTGCGGCGTCTTTGTCGTGGACGGCACGCGGGAGCTGGTTAGGCAATTGGCGCAAGGATGGGAGCCAGACCAGCTGAACCTTAGCAACGTCGTTGCCAATCGGCAGGCGCTGCAGAACCGACTCAGGGGT**CCCCGGGGATCCTCTAGAGTCGACCTGCAGGATTACAAGGATGACGACGATAAGGACTATAAGGACGATGATGACAAGGACTACAAAGATGATGACGATAAATAGGCATGCAAGCTTGGCACTGGTACCCAATTCGCCCTAGCCCG

**(C) *Δbel2-5::bel2-5_xopDULP1***

***﻿***CTAAAGGGAACAAAAGCTGGAGCTCGGGGTGACGTGGGTGTCATTCTGCAATTCTGCCCCCCTAAGCCGGGGGATCGGCGTCCAAAATTTGCAAGCCGATTCAGCAATAGGCGTCTTAAGATCAATACTGAATTGTAAGGCACCAAAAGTGGTTGCGTTTCGATTTTTGCCTCGCGCGTTCAGCGCATCCTGTCAAGATGTTGGCTGGAGCCGCGAAAGCTGGCCGTACGCCTCACCGATGAGAAGCAGTATTTTGTCTCTGAATCTTCGGTATCTTCGGTCTCTCGGCTGTTGGCGGCGAAGCGCGCAGGCACATGCACCATGCTGTCTTGGCGCGGCAATTTTCGTCATCAGGTTCTCGACAGCTTCCCCATTTAACAAAACATACAGGGCTGGTCGCCTTGCAAAAACATCGGCTGCCGATGCTCACGGTGCGCGCCTGTCGAGGGCCGTAAAGGGTGGTTTGTTGCTGATTCCCAGCATGTCAGATCGAAGCCCATGTTCATATTTTGAAGGTGAGAACGG**ATGGATTTCCCCTCGACCAAGTCGGTGCATGAACAATCGGACAGCACGGGCCCGCAAGAGAGCTCACCCGCGGCTCCGTCCGCAGCCGCGGCGACCTTTGAGCGGCAATTGAGCGAGATCGCCAACTCAGATGGAGGTGGTGGAGCAATGCCGGCCGCCTCGGCGCTGCAGTCGGCTCAGTCAACTTGGGTTTTGATAGGACGGCAGGGCAAGCATCTCCTTTATTCCGAGGACGCTCGCCTTATTTCGGGGCTTGAGAAGGCCCTTATCGGCAACCCCACCCACCGCAACGCTGGGGACTATCTAACTTCACTTCGCAGCTTTGGCGGCTGGCTCTTCGCAAATAACAAAACGAGCATTGTTGCTCGGCTCAACGACCCGTCGCTGACCCATGATGCACGCGAGTTCGAAAAGAGGCGTCCCTCGAATATCCTTGCGGCATTGAACCATCTCCGGACCTTCCAGTCGACGGGCGGAGTGACGGTCACAGCCCGCACTGAGCTAAATCCTCACCCTCAGGACGCGGATCTCATCAACGAGTACAAAAAAGAAACAGCGACAAGTACCGGCAGGATGTATGCAACTGCTCTTCGCAGTTTCGGTCACTACCTGCGTGAAAACAACAAGAAGGGCATTGCTACTCGGCTTTCCGGCGGGGCGTTGGATGAAGATGTCAATAGCTATAAGAAGGGCGCCGGTGCTGATTCGAGGATCGGTGCCGCTCTGGCTCAACTCCGAAAATCGCAGGCCGGCGCTAAGGCGATGGAGCCCGAGCGCCATTTTGATCCCAAAGACGCGGCCCTGATGGAGTCGATGCAGGTCGGCGACGCCGCTGCGCAGCACAGTGCGTCGCAGCAAGCTGGCAGTTGGTCAGAGGAATTGAACCATCTCCGGACCTTCCAGTCGACGGGCGGAGTGACGGTCACAGCCCGCACTGAGCTAAATCCTCACCCTCAGGACGCGGATCTCATCAACGAGTACAAAAAAGAAACAGCGACAAGTACCGGCCGGATGTATGCAACTGCTCTTCGCAGTTTCGGTCACTACCTGCGTGAAAATAACAAGAAGGGCATTGCTACTCGGCTTTCCGGCGGGGCGTTGGATGAAGATATCAATAGCTATAAGAAGGGCGCCGGTGCTGATTCGAGGATCGGTGCCGCTCTGGCTCAACTCCGAAAATCGCAGGCCGGCGCTAACGCGATGGAGCTCGAGCGGCATATTGATCCCAAAGACGCGGCCCTGATGGAGTCGATGCAGGTCGGCGACGCCGCTGCGCAGCACAGTGCGTCGCAGCAAGCTGGCAGTTGGCCAGAGGAATTTCTTCCTGCGGAAGGCCACGATCAGGATTTGGGCCGGATGGACGAACCCGGCCCGTCGTCGTCGGCGCCGCAGCCGGCTCAGTCAACTGGGATTTTGAGAGGGCGGAGGAAGCCTCTTTATTCCGAGGATGCTCCCCTTATTTCGGGGCTTGAGGAGGCCCTCCGCAGTGGCAACGCCGCCGAACGCACCGCCAAAGACCTTGTAGGCCCGCTTCGCGCCTTTGGCCGCTGGCTCTTGGCAAATAACAAAACGAGCATTGTTGATCGGCTCGAAAAAGAGTCGCTGACCGATGATGCGCGTGAGTTCATCGAAAAGGGTAAGGGCAGTCGCCTCCTTATTAGGTCAATAGGTCTTCTCCGGACCTTTCAGTCGACGGGCGGAGTGACGGTCACAGCCACCACTGAGCTAAATCCTTACCTCCAGGACGCGGCTCTCATCATAGAGTACCAAAACGAAGCAGCGACAAGTACCGGCGGTGTGTATGCGACTGCTCTTCGCAGTTTCGGTCACCACCTGCGTGAAAATAACAAGAAGGGCATTGCTACTCGGCTTTCCGGCGGGGCGTTGGATGAAGATGTCGAGGCCTATAAAAAGGACTTCGGTGGCATCCGGACGATCGATGCCGCATTAGGTCAACTCCGAAAATCACAGGCGGGCGCTAAAGCGATGGAGCGCGAGCGCTATATTTCCCCCGGTCCTGATCCCGAAGGCGCGGCACTGATGGAGCCGAGGCGGGCCGGCGACGCCGCAGCGCAGCACAGCTCGCAGGAAGTTGGCAGTTGGCCAGAGAAACTGCTTCCTGCGGAACGCCACGAGCAGGATTTGGTTTTGGGGCTGATGGACGAACCCGGCCCGTCGTCATCTCTCGAGCCAGTCGCGCGGCACGACCAGGCATCGGATCCCGGAGATTCCATTCGTCCCCTGAACTGGCGCCGGGACGGCCAGCAGTTCTCGGAAGAGCCGATGGCTGCACTTGCCAGGAGCAACCTCCCGCCAAGCGAGGAGATCCTCATCAACGATGAACAGGATGCAGCTGAGTTAAGGCCAGCGAAGAGGCCGAGGACCCTAGACAATCCGCAAGGCCTTGCTATTGAGCGGCTGCTGAGCGAGATCGCCGCGACCCCGGCCCCCACCCATCAACAGGGTGCATCGCCATGGCATGCGCAGCCGATGATGCAGGCGAGCGGGCACGAAGATGCAACGGCGCCGCATGCGGCCGCGACGTACGTCGCGGGCGCCGCCGCGCAGCACAGCGCGCCGCAGGGAGCTGTCAGTCGGCCATTGGTCCTCCCGGAAGGTTACGACCGGGATCTGCGTTTGATGGGGAAAGACGGCCCATCGTGGCCCGAGGTTCCCCCTGGGCAGGCGCAGGACATAGTCCAAGCTGGACGGCAGCAACCTGCGTGGTCCGCCTCAACCTGGTCGCCGCAGATGCCGCTCGACTTTGATTGGAGTATGTGGCCGACGCTGGAAGCAGCGCCGGCGCCGGCTGCCAGGGCTCGCTCAGGCACCTACGGCGGTCTTGAGTCATTGGTGCATCTGGATGCGCCCACGCCGTCCGAATTGCGCGACGATGCTCACTTTGCGCCGGCGCCCGCTGCCAGGGCTCGCTCAGGCACCTACGGCGGTCTTGAGTCATTGGTGCATCTGGATGCGCCCACGCCGTCCGAATTGCGCGACGATGCTCACTTTGCGCCGGCGCCCGCTGCCAGGGCTCGCTCAGACACCTACGGCGGTCTTGAGTCATTGGTGCATCTGGATGCGCCCACGCCGTCCGAATTACGCGACGATGCTCACTTTGCGCCGGCGCCCGCTGCCAGGGCTCGCTCAGACACCTACGGCGGTCTTGAGTCATTGGTGCATCTGGATGCGCCCACGCCGTCCGAGTTGCGCGACGATGCTCACTTTGCGGCGGCGCCTTTTGCCAGGGCTCGCTCAGACGCCTACCGCGGTTTTCCATTGGTCGATCTGACTGCGCCCACGCCGTCCGAATCACGTGACGATGCTAATTCTGTACGCCCGTTTCCGAGCACCTCCGCTAATGCTCAGATCGGGGCTTTAGATCCGACAGTCTCGTCTCACGGCCACGGGCTGGTGCTCGATGACACTTCGTGGCTGCTGGATGGACATTTGCGCGCCTACACCGATGACCTAGCTCGCCGATTGCGAGGGGAGCCCAACGCCCATTTACTCCACTTTGCCGACTCGCAGGTAGTGACCATGCTGAGCTCCGCAGATCCAGACCAACAGGCCCGCGCACAGCGCCTTCTTGCCGGAGACGACATCCCACCTATCGTGTTCCTGCCGATCAATCAGCCCAACGCTCATTGGTCATTGCTCGTCGTCGACCGGCGTAACAAGGACGCTGTTGCGGCCTACCACTATGATTCCATGGCACAGAAGGACCCACAGCAACGCTACCTTGCTGATATGGCGGCCTATCACCTTGGCCTTGATTATCAACAAACTCATGAAATGCCCATCGCGATACAGTCGGACGGTTATTCCTGCGGCGATCATGTGCTGACCGGGATAGAGGTGTTGGCCCACAGGGTACTCGACGGCACCTTCGACTACGCAGGCGGCAGGGACCTGACTGATATCGAACCAGACCGCGGCCTCATCAGGGATCGTCTTGCCCAAGCGGAGCAAGCTCCAGCAGAAAGCAGCATCAGGCAAGTTCCCGCACGATCCAACGAACAGAAGAAAAAGAAAAGCAAGTGGTGGAAAAAGTTC**CCCCGGGGATCCTCTAGAGTCGACCTGCAGGATTACAAGGATGACGACGATAAGGACTATAAGGACGATGATGACAAGGACTACAAAGATGATGACGATAAATAGGCATGCAAGCTTGGCACTGGTACCCAATTCGCCCTAGCCCG

**Supplementary Data S3.** Alignment of Bel2-5 effector and its homologs in *B. japonicum* USDA110, including Bll8244 (BAC53509.1), Blr1693 (BAC46958.1), and Blr1705 (BAC46970.1) using the Praline multiple sequence alignment program (http://www.ibi.vu.nl).

**
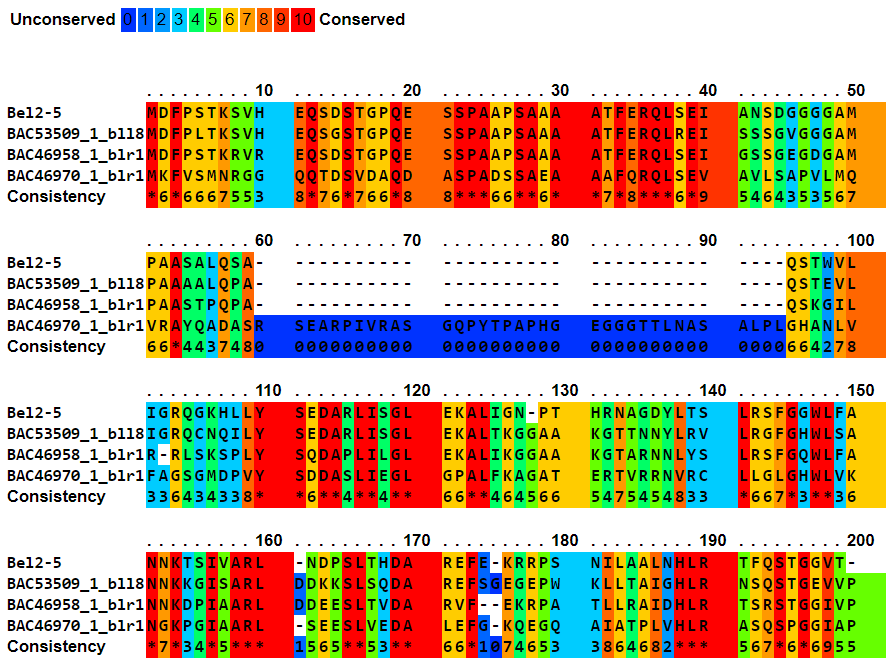
**

Bel2-5

Bll8244

Blr1693

Blr1705

Consistency

Bel2-5

Bll8244

Blr1693

Blr1705

Consistency

Bel2-5

Bll8244

Blr1693

Blr1705

Consistency

Bel2-5

Bll8244

Blr1693

Blr1705

Consistency

Bel2-5

Bll8244

Blr1693

Blr1705

Consistency

**
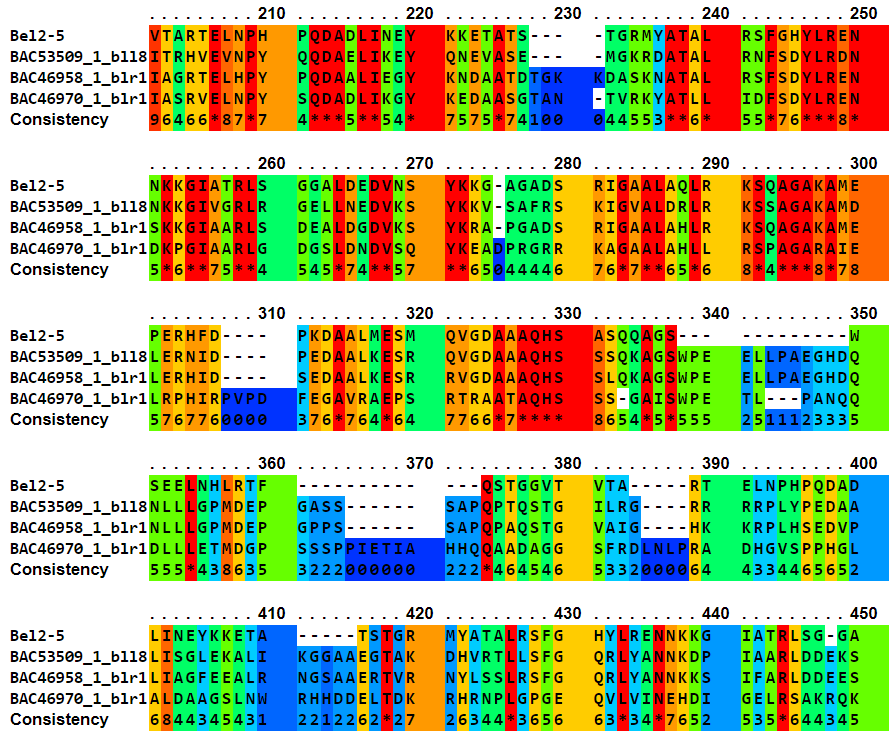
**

Bel2-5

Bll8244

Blr1693

Blr1705

Consistency

Bel2-5

Bll8244

Blr1693

Blr1705

Consistency

Bel2-5

Bll8244

Blr1693

Blr1705

Consistency

Bel2-5

Bll8244

Blr1693

Blr1705

Consistency

**
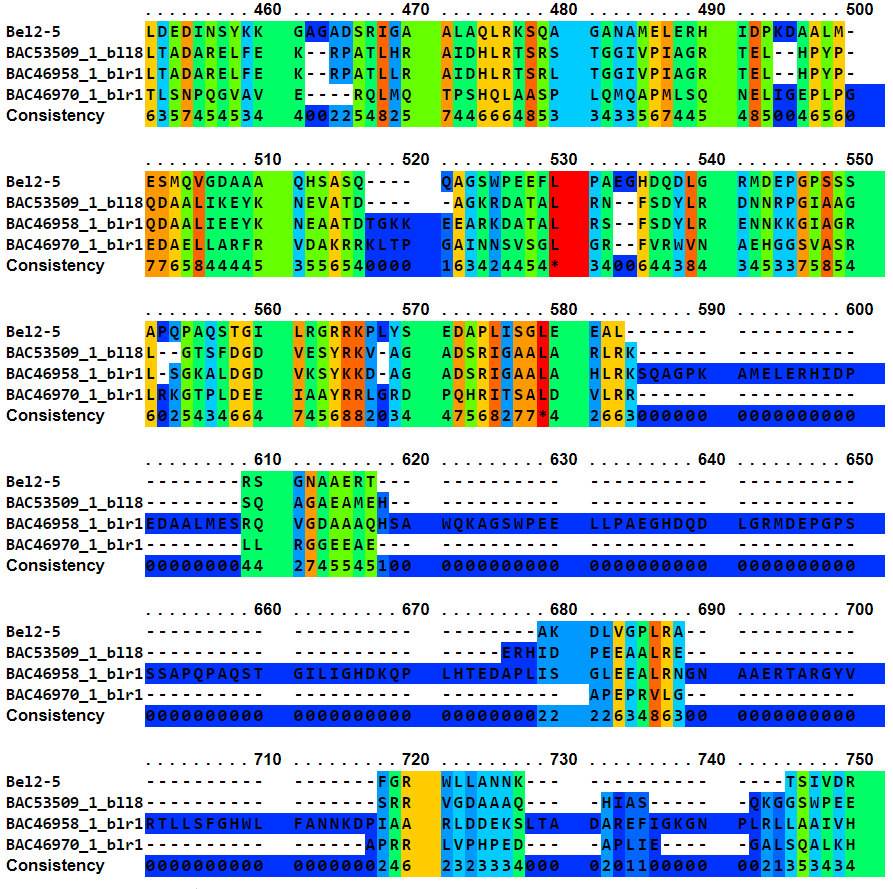
**

Bel2-5

Bll8244

Blr1693

Blr1705

Consistency

Bel2-5

Bll8244

Blr1693

Blr1705

Consistency

Bel2-5

Bll8244

Blr1693

Blr1705

Consistency

Bel2-5

Bll8244

Blr1693

Blr1705

Consistency

Bel2-5

Bll8244

Blr1693

Blr1705

Consistency

Bel2-5

Bll8244

Blr1693

Blr1705

Consistency

Bel2-5

Bll8244

Blr1693

Blr1705

Consistency

Bel2-5

Bll8244

Blr1693

Blr1705

Consistency

Bel2-5

Bll8244

Blr1693

Blr1705

Consistency

Bel2-5

Bll8244

Blr1693

Blr1705

Consistency

**
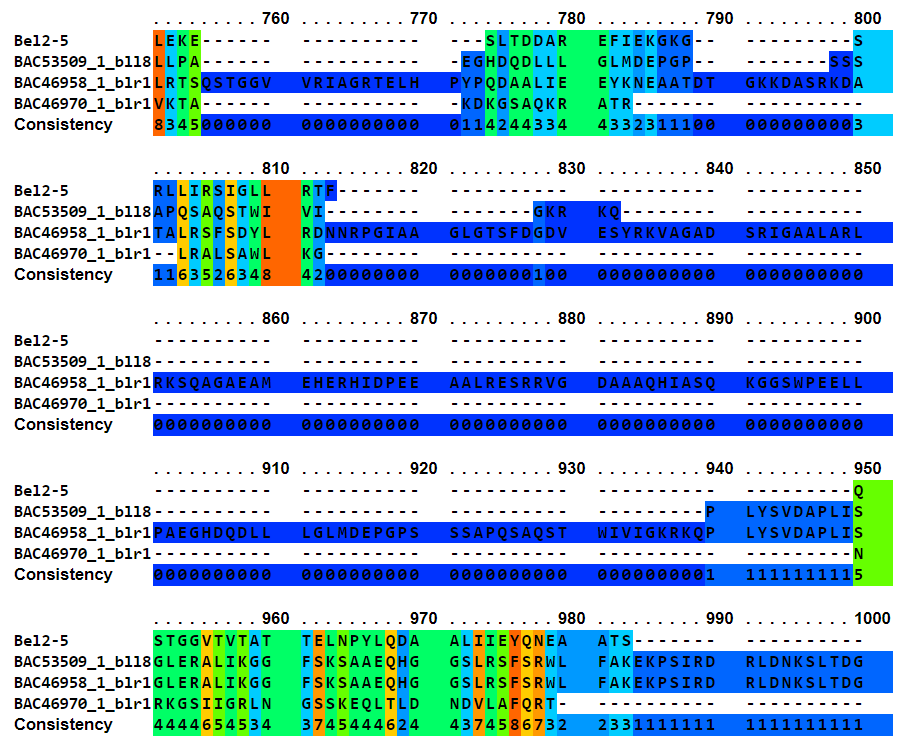
**

Bel2-5

Bll8244

Blr1693

Blr1705

Consistency

**
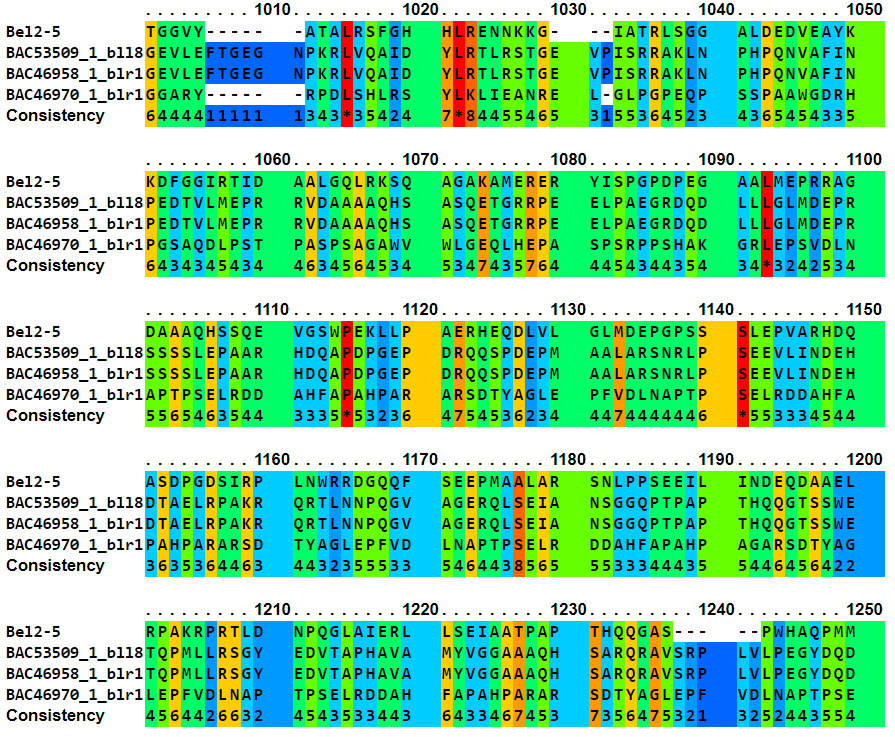
**

Bel2-5

Bll8244

Blr1693

Blr1705

Consistency

Bel2-5

Bll8244

Blr1693

Blr1705

Consistency

Bel2-5

Bll8244

Blr1693

Blr1705

Consistency

Bel2-5

Bll8244

Blr1693

Blr1705

Consistency

Bel2-5

Bll8244

Blr1693

Blr1705

Consistency

**
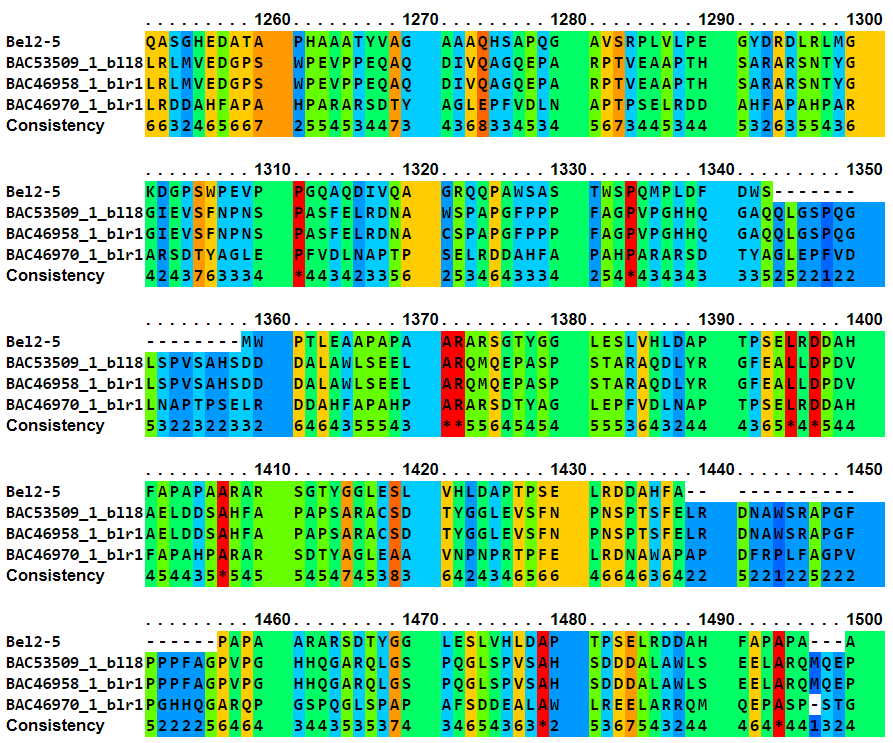
**

Bel2-5

Bll8244

Blr1693

Blr1705

Consistency

Bel2-5

Bll8244

Blr1693

Blr1705

Consistency

Bel2-5

Bll8244

Blr1693

Blr1705

Consistency

Bel2-5

Bll8244

Blr1693

Blr1705

Consistency

Bel2-5

Bll8244

Blr1693

Blr1705

Consistency

# Supplementary Figures and Tables

**2.1 Supplementary Figures**


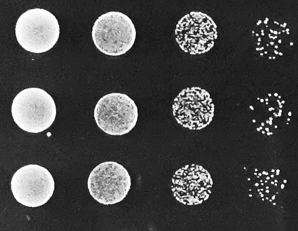

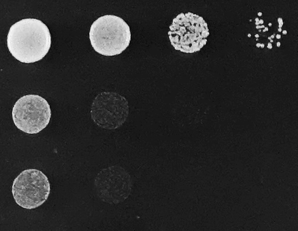


10^0^

10^-1^

10^-2^

10^-3^

10^0^

10^-1^

10^-2^

10^-3^

Dilution:

Empty vector

Bel2-5

Bel2-5-GFP

*P_GAL1_* expression:

ON

OFF

**Supplementary Figure S1.** GFP-tagging at the C-terminus of Bel2-5 did not affect its yeast growth inhibitory activity. Expression of the GFP-tagged or untagged Bel2-5 effector. Bel2-5 expression was under the control of the inducible promoter *GAL1*.

**2.2**  **Supplementary Tables**

**Supplementary Table S1.** Soybean seeds, bacterial or yeast strains, oligonucleotides, and plasmids used in this study

| **Strain** | **Characteristics or sequence** | **Construction** | **Reference or source** |
| --- | --- | --- | --- |
| Soybean seeds |  |  |  |
| *Glycine max* |  |  |  |
| En1282 | Non-nodulating mutant of Enrei derived by EMS treatment, carrying mutant alleles of *GmNFR1* |  | (Francisco and Akao, 1993; Ikeda et al., 2008) |
| BARC-2 | Carrying *Rj4* allele |  | (Devine and O`Neill, 1986) |
| Bacterial strains |  |  |  |
| *Bradyrhizobium elkanii* |  |  |  |
| USDA61 | Wild-type stain, Pol^r^ |  | Keyser^b^ |
| *∆bel2-5* | USDA61 derivate with a deletion of *bel2-5* gene, Pol^r^ |  | (Ratu et al., 2021) |
| BEC1286A | USDA61 derivate carrying an amino acid substitution of Cys (C1286) with Ala in the ULP-like domain of Bel2-5, Pol^r^, Sm^r^, Sp^r^ |  | (Ratu et al., 2021) |
| BERD1 | *∆bel2-5* complemented with *bel2-5* gene carrying an amino acid deletion of the predicted repeat domain I (Δ148-438aa) of Bel2-5, Pol^r^, Sm^r^, Sp^r^ | a ~4.5-kb DNA fragment containing *bel2-5* with its promoter sequence was synthesized and cloned into the carrier plasmid pUC57, which was then used as the template for generating a plasmid carrying a deletion of the predicted repeat domain I (Δ148-438aa) using QuikChange Lightning Site-Directed Mutagenesis Kit (Agilent Technologies, CA, USA) with specific primer Bel2-5_DelRepeatI_R/F. The constructed plasmid was confirmed using Sanger sequencing. Next, the *bel2-5* sequence with desired point mutation was used as the template for further amplification using specific primer pBjGroEL4::*bel2-5*_F/R to create *SacI*/*KpnI* sites for cloning into plasmid pBjGroEL4::dsRED. The resultant DNA fragment was integrated into the *bel2-5* deletion mutant (*∆bel2-5*) through single-crossover recombination. The constructed mutant was screened for antibiotic resistance and confirmed by PCR and sequence analysis. | This study |
| BEEAR1 | *∆bel2-5* complemented with *bel2-5* gene carrying an amino acid substitution of the predicted EAR motif I (from LVLGL into AVAGA) of Bel2-5, Pol^r^, Km^r^ | a ~4.5-kb DNA fragment containing *bel2-5* with its promoter sequence was synthesized and cloned into the carrier plasmid pUC57, which was then used as the template for generating a plasmid carrying a substitution of the predicted EAR motif 1 (715-719aa) using QuikChange Lightning Site-Directed Mutagenesis Kit (Agilent Technologies, CA, USA) with specific primer Bel2-5_EAR1-subt_R/F. The constructed plasmid was confirmed using Sanger sequencing. Next, the *bel2-5* sequence with desired point mutations was excised from the carrier plasmid (pUC57) by digestion at the *EcoRI*/*XbaI* sites and transferred into plasmid pK18mob. The resultant DNA fragment was integrated into the *bel2-5* deletion mutant (*∆bel2-5*) through single-crossover recombination. The constructed mutant was screened for antibiotic resistance and confirmed by PCR and sequence analysis. | This study |
| BENLS | *∆bel2-5* complemented with *bel2-5* gene carrying an amino acid substitution of the predicted NLS motif (from RPAKRPRTL 🡪 APAGAPATL) of Bel2-5, Pol^r^, Sm^r^, Sp^r^ | a ~4.5-kb DNA fragment containing *bel2-5* with its promoter sequence was synthesized and cloned into the carrier plasmid pUC57, which was then used as the template for generating a plasmid carrying a substitution of the predicted NLS (788-796aa) using QuikChange Lightning Site-Directed Mutagenesis Kit (Agilent Technologies, CA, USA) with specific primer Bel2-5_NLS-subt_R/F. The constructed plasmid was confirmed using Sanger sequencing. Next, the *bel2-5* sequence with desired point mutation was used as the template for further amplification using specific primer pBjGroEL4::*bel2-5*_F/R to create *SacI*/*KpnI* sites for cloning into plasmid pBjGroEL4::dsRED. The resultant DNA fragment was integrated into the *bel2-5* deletion mutant (*∆bel2-5*) through single-crossover recombination. The constructed mutant was screened for antibiotic resistance and confirmed by PCR and sequence analysis. | This study |
| BERD2 | *∆bel2-5* complemented with *bel2-5* gene carrying an amino acid deletion of the predicted repeat domain II (Δ932-1109aa) of Bel2-5, Pol^r^, Km^r^ | a ~4.5-kb DNA fragment containing *bel2-5* with its promoter sequence was synthesized and cloned into the carrier plasmid pUC57, which was then used as the template for generating a plasmid carrying a deletion of the predicted repeat domain 2 (Δ932-1109aa) using QuikChange Lightning Site-Directed Mutagenesis Kit (Agilent Technologies, CA, USA) with specific primer Bel2-5_DelRepeat2_R/F. The constructed plasmid was confirmed using Sanger sequencing. Next, the *bel2-5* sequence with desired point mutation was excised from the carrier plasmid (pUC57) by digestion at the *EcoRI*/*XbaI* sites and transferred into plasmid pK18mob. The resultant DNA fragment was integrated into the *bel2-5* deletion mutant (*∆bel2-5*) through single-crossover recombination. The constructed mutant was screened for antibiotic resistance and confirmed by PCR and sequence analysis. | This study |
| BEEAR2 | *∆bel2-5* complemented with *bel2-5* gene carrying an amino acid substitution of the predicted EAR motif II (from LNLPLEL 🡪 ANAPAEA) of Bel2-5, Pol^r^, Sm^r^, Sp^r^ | a ~4.5-kb DNA fragment containing *bel2-5* with its promoter sequence was synthesized and cloned into the carrier plasmid pUC57, which was then used as the template for generating a plasmid carrying a substitution of the predicted EAR motif 2 (1268-1274aa) using QuikChange Lightning Site-Directed Mutagenesis Kit (Agilent Technologies, CA, USA) with specific primer Bel2-5_EAR2-subt_R/F. The constructed plasmid was confirmed using Sanger sequencing. Next, the *bel2-5* sequence with desired point mutation was used as the template for further amplification using specific primer pBjGroEL4::*bel2-5*_F/R to create *SacI*/*KpnI* sites for cloning into plasmid pBjGroEL4::dsRED. The resultant DNA fragment was integrated into the *bel2-5* deletion mutant (*∆bel2-5*) through single-crossover recombination. The constructed mutant was screened for antibiotic resistance and confirmed by PCR and sequence analysis. | This study |
| *Δbel2-5::xopD* | *∆bel2-5* complemented with the full-length of *Xcv*. XopD, *bel2-5* promoter on its upstream region and 3xFLAG sequence on C-terminus, Pol^r^, Sm^r^, Sp^r^ | a DNA fragment containing full length of *Xcv.* XopD and *bel2-5* promoter was artificial synthesized and cloned into carrier plasmid pUC57. The synthetic sequence then was used as the template for PCR amplification using specific primer sets for cloning into plasmid pBjGroEL4::dsRED at *SacI*/*KpnI* sites and confirmed by Sanger sequencing. The constructed plasmid was then integrated into the *bel2-5* deletion mutant (*∆bel2-5*) through single homologous recombination. The constructed *B. elkanii* mutant strain was screened for antibiotic resistance and confirmed by PCR. | This study |
| *Δbel2-5::xopD_bel2-5ULP* | *∆bel2-5* complemented with *xopD* harbouring ULP domain of *bel2-5*, *bel2-5* promoter on its upstream region, and 3xFLAG sequence on C-terminus, Pol^r^, Sm^r^, Sp^r^ | a DNA fragment containing partial sequence of *Xcv.* XopD harbouring ULP-like domain of Bel2-5 in C-terminus and *bel2-5* promoter was artificial synthesized and cloned into carrier plasmid pUC57. The synthetic sequence then was used as the template for PCR amplification using specific primer sets for cloning into plasmid pBjGroEL4::dsRED at *SacI*/*KpnI* sites and confirmed by Sanger sequencing. The constructed plasmid was then integrated into the *bel2-5* deletion mutant (*∆bel2-5*) through single homologous recombination. The constructed *B. elkanii* mutant strain was screened for antibiotic resistance and confirmed by PCR. | This study |
| *Δbel2-5::bel2-5_xopDULP* | *∆bel2-5* complemented with *bel2-5* harbouring ULP domain of *xopD*, *bel2-5* promoter on its upstream region, and 3xFLAG sequence on C-terminus, Pol^r^, Sm^r^, Sp^r^ | a DNA fragment containing partial sequence of Bel2-5 harbouring ULP-like domain of *Xcv*. XopD in C-terminus and *bel2-5* promoter was artificial synthesized and cloned into carrier plasmid pUC57. The synthetic sequence then was used as the template for PCR amplification using specific primer sets for cloning into plasmid pBjGroEL4::dsRED at *SacI*/*KpnI* sites and confirmed by Sanger sequencing. The constructed plasmid was then integrated into the *bel2-5* deletion mutant (*∆bel2-5*) through single homologous recombination. The constructed *B. elkanii* mutant strain was screened for antibiotic resistance and confirmed by PCR. | This study |
| *Escherichia coli* |  |  |  |
| DH10B | Cloning strain |  | Invitrogen, CA, USA |
| HB101 | *recA hsdR hsdM pro* Smr |  | Invitrogen, CA, USA |
| S17 | *hsdR pro thi* (RP4-2 *km*::Tn*7 Tc*::Mu integrated into the chromosome) Smr Spr |  | (Simon et al., 1983) |
| *Saccharomyces cerevisiae* | | | |
| MTY914 | *smt3*Δ::*KanMX4* carrying pRS415-HA-*SMT3* | The *SMT3* promoter region, *SMT3* ORF and *SMT3* 3’-UTR region sequences were amplified using specific primer sets MTO1156/MTO1157 followed by amplification of *att*B-adaptor primers MTO529/MTO530 and cloned into pDONR221 by the BP reaction, resulting in pDONR221-*SMT3*. The N-terminal HA-tagging into *SMT3* gene was introduced by inverse PCR using a specific primer set, MTO1154/MTO1155, resulting pDONR221-HA-*SMT3*. The entry clone was screened for antibiotic resistance and confirmed by PCR and sequence analysis. The pDONR221-HA-*SMT3* was subcloned into gateway vector pMT1271 via LR reaction, resulting in pMT1487. The pMT1487 was transformed into heterozygote *SMT3* knockout strain (α/a *smt3*Δ::*KanMX4*/*SMT3)* and transformant was sporulated and haploid *smt3*Δ::*KanMX4* carrying pRS415-HA-*SMT3* cell was obtained by tetrad-dissection analysis. | This study |
| Bel2-5 Wild-type (WT) | Yeast MTY914 expressing the full-length sequence of Bel2-5, *URA3* (2μ) | The full length of Bel2-5 sequence without stop codon was amplified using a specific primer set, Bel2-5GWF/Bel2-5GWR1 followed by amplification of *att*B-adaptor primers MTO529/MTO530　and cloned into pDONR207 by the BP reaction, resulting in pAH64. The entry clone was screened for antibiotic resistance and confirmed by PCR and sequence analysis. The pAH64 was subcloned into gateway vector pMT751 via LR reaction, resulting in pAH66. The pAH66 was transformed into MTY914 (*smt3*Δ::*KanMX4* carrying pRS415-HA-*SMT3*). | This study |
| Δ2-438 aa | Yeast MTY914 expressing the partial sequence of Bel2-5 with deletion in Repeat Domain 1, *URA3* (2μ) | The full length of Bel2-5 sequence without stop codon was amplified using a specific primer set, Bel2-5GWF/Bel2-5GWR1 followed by amplification of *att*B-adaptor primers MTO529/MTO530 and cloned into pDONR207 by the BP reaction, resulting in pAH64. The pAH64 was used as a template for generating plasmid pAH103, which carrying deletion on the predicted repeat domain 1 (Δ2-438 aa) sequence. The partial deletion of Bel2-5 was performed by inverse PCR with specific primer sets AHO85/AHO97 and confirmed by sequence analysis. The pAH103 was subcloned into gateway vector pMT751 via LR reaction, resulting in pAH111. The pAH111 was transformed into MTY914 (*smt3*Δ::*KanMX4* carrying pRS415-HA-*SMT3*). | This study |
| Δ2-725 aa | Yeast MTY914 expressing the partial sequence of Bel2-5 with deletion in Repeat Domain 1 and EAR motif 1, *URA3* (2μ) | The full length of Bel2-5 sequence without stop codon was amplified using a specific primer set, Bel2-5GWF/Bel2-5GWR1 followed by amplification of *att*B-adaptor primers MTO529/MTO530 and cloned into pDONR207 by the BP reaction, resulting in pAH64. The pAH64 was used as a template for generating plasmid pAH104, which carrying deletion on the predicted repeat domain 1 and EAR motif 1 (Δ2-725 aa) sequence. The partial deletion of Bel2-5 was performed by inverse PCR with specific primer sets AHO85/AHO98 and confirmed by sequence analysis. The pAH104 was subcloned into gateway vector pMT751 via LR reaction, resulting in pAH112. The pAH112 was transformed into MTY914 (*smt3*Δ::*KanMX4* carrying pRS415-HA-*SMT3*). | This study |
| Δ2-796 aa | Yeast MTY914 expressing the partial sequence of Bel2-5 with deletion in Repeat Domain 1, EAR motif 1, and NLS, *URA3* (2μ) | The full length of Bel2-5 sequence without stop codon was amplified using a specific primer set, Bel2-5GWF/Bel2-5GWR1 followed by amplification of *att*B-adaptor primers MTO529/MTO530 and cloned into pDONR207 by the BP reaction, resulting in pAH64. The pAH64 was used as a template for generating plasmid pAH105, which carrying deletion on the predicted repeat domain 1, EAR motif 1, and NLS (Δ2-796aa) sequence. The partial deletion of Bel2-5 was performed by inverse PCR with specific primer sets AHO85/AHO98 and confirmed by sequence analysis. The pAH105 was subcloned into gateway vector pMT751 via LR reaction, resulting in pAH113. The pAH113 was transformed into MTY914 (*smt3*Δ::*KanMX4* carrying pRS415-HA-*SMT3*). | This study |
| Δ2-1109 aa | Yeast MTY914 expressing the partial sequence of Bel2-5 with deletion in Repeat Domain 1, EAR motif 1, NLS, and Repeat Domain 2, *URA3* (2μ) | The full length of Bel2-5 sequence without stop codon was amplified using a specific primer set, Bel2-5GWF/Bel2-5GWR1 followed by amplification of *att*B-adaptor primers MTO529/MTO530 and cloned into pDONR207 by the BP reaction, resulting in pAH64. The pAH64 was used as a template for generating plasmid pAH106, which carrying deletion on the predicted repeat domain 1, EAR motif 1, NLS, and repeat domain 2 (Δ2-1109 aa) sequence. The partial deletion of Bel2-5 was performed by inverse PCR with a specific primer set, AHO85/AHO98 and confirmed by sequence analysis. The pAH106 was subcloned into gateway vector pMT751 via LR reaction, resulting in pAH114. The pAH114 was transformed into MTY914 (*smt3*Δ::*KanMX4* carrying pRS415-HA-*SMT3*). | This study |
| 1-1143 aa | Yeast MTY914 expressing the partial sequence of Bel2-5 carrying Repeat Domain 1, EAR motif 1, NLS, and Repeat Domain 2, *URA3* (2μ) | The full length of Bel2-5 sequence without stop codon was amplified using primers Bel2-5GWF/Bel2-5GWR1 followed by amplification of *att*B-adaptor primers MTO529/MTO530 and cloned into pDONR207 by the BP reaction, resulting in pAH64. The pAH64 was used as a template for generating plasmid pAH107, which carrying the predicted repeat domain 1, EAR motif 1, NLS, and repeat domain 2 (1-1143aa) sequence. The partial deletion of Bel2-5 was performed by inverse PCR with a specific primer set, JHO18/AHO101 and confirmed by sequence analysis. The pAH107 was subcloned into gateway vector pMT751 via LR reaction, resulting in pAH115. The pAH115 was transformed into MTY914 (*smt3*Δ::*KanMX4* carrying pRS415-HA-*SMT3*). | This study |
| 1-931 aa | Yeast MTY914 expressing the partial sequence of Bel2-5 carrying Repeat Domain 1, EAR motif 1, and NLS, *URA3* (2μ) | The full length of Bel2-5 sequence without stop codon was amplified using primers Bel2-5GWF/Bel2-5GWR1 followed by amplification of *att*B-adaptor primers MTO529/MTO530 and cloned into pDONR207 by the BP reaction, resulting in pAH64. The pAH64 was used as a template for generating plasmid pAH108, which carrying the predicted repeat domain 1, EAR motif 1, and NLS motif (1-931aa) sequence. The partial deletion of Bel2-5 was performed by inverse PCR with a specific primer set, JHO18/AHO102 and confirmed by sequence analysis. The pAH108 was subcloned into gateway vector pMT751 via LR reaction, resulting in pAH116. The pAH116 was transformed into MTY914 (*smt3*Δ::*KanMX4* carrying pRS415-HA-*SMT3*). | This study |
| 1-787 aa | Yeast MTY914 expressing the partial sequence of Bel2-5 carrying Repeat Domain 1 and EAR motif 1, *URA3* (2μ) | The full length of Bel2-5 sequence without stop codon was amplified using primers Bel2-5GWF/Bel2-5GWR1 followed by amplification of *att*B-adaptor primers MTO529/MTO530 and cloned into pDONR207 by the BP reaction, resulting in pAH64. The pAH64 was used as a template for generating plasmid pAH109, which carrying the predicted repeat domain 1 and EAR motif 1 (1-787aa) sequence. The partial deletion of Bel2-5 was performed by inverse PCR with a specific primer set, JHO18/AHO103 and confirmed by sequence analysis. The pAH109 was subcloned into gateway vector pMT751 via LR reaction resulting in pAH117. The pAH117 was transformed into MTY914 (*smt3*Δ::*KanMX4* carrying pRS415-HA-*SMT3*). | This study |
| 1-714 aa | Yeast MTY914 expressing the partial sequence of Bel2-5 carrying Repeat Domain I, *URA3* (2μ) | The full length of Bel2-5 sequence without stop codon was amplified using primers Bel2-5GWF/Bel2-5GWR1 followed by amplification of *att*B-adaptor primers MTO529/MTO530 and cloned into pDONR207 by the BP reaction, resulting in pAH64. The pAH64 was used as a template for generating plasmid pAH110, which carrying the predicted repeat domain 1 (1-714aa) sequence. The partial deletion of Bel2-5 was performed by inverse PCR with specific primer sets JHO18/AHO104 and confirmed by sequence analysis. The pAH110 was subcloned into gateway vector pMT751 via LR reaction, resulting in pAH118. The pAH118 was transformed into MTY914 (*smt3*Δ::*KanMX4* carrying pRS415-HA-*SMT3*). | This study |
| NLS | Yeast MTY914 expressing the full-length sequence of Bel2-5 with an amino acid substitution on predicted NLS motif (from RPAKRPRTL 🡪 APAGAPATL), *URA3* (2μ) | The full length of Bel2-5 sequence carrying an amino acid substitution on NLS motif (from RPAKRPRTL 🡪 APAGAPATL) without stop codon was amplified using a specific primer set, Bel2-5GWF/Bel2-5GWR1 followed by amplification of *att*B-adaptor primers MTO529/MTO530 and cloned into pDONR207 by the BP reaction, resulting in pAH70. The entry clone was confirmed by sequence analysis. The pAH70 was subcloned into gateway vector pMT751 via LR reaction, resulting in pAH72. The pAH72 was transformed into MTY914 (*smt3*Δ::*KanMX4* carrying pRS415-HA-*SMT3*). | This study |
| C1286A | Yeast MTY914 expressing the full-length sequence of Bel2-5 with an amino acid substitution on putative catalytic site of ULP-like domain, cysteine into alanine, *URA3* (2μ) | The full length of Bel2-5 sequence carrying an amino acid substitution on catalytic site of ULP-like domain (from cysteine to alanine, C1286A) without stop codon was amplified using a specific primer set, Bel2-5GWF/Bel2-5GWR1 followed by amplification of *att*B-adaptor primers MTO529/MTO530 and cloned into pDONR207 by the BP reaction, resulting in pAH69. The entry clone was confirmed by sequence analysis. The pAH69 was subcloned into gateway vector pMT751 via LR reaction, resulting in pAH71. The pAH72 was transformed into MTY914 (*smt3*Δ::*KanMX4* carrying pRS415-HA-*SMT3*). | This study |
| Δ2-1109 aa (C1286A) | Yeast MTY914 expressing the partial sequence of Bel2-5 with deletion in Repeat Domain 1, EAR motif 1, NLS, and Repeat Domain 2 and carrying an amino acid substitution on putative catalytic site of ULP-like domain, cysteine into alanine (C1286A), *URA3* (2μ) | The full length of Bel2-5 sequence carrying an amino acid substitution on catalytic site of ULP-like domain (from cysteine to alanine, C1286A) without stop codon was amplified using a specific primer set, Bel2-5GWF/Bel2-5GWR1 followed by amplification of *att*B-adaptor primers MTO529/MTO530 and cloned into pDONR207 by the BP reaction, resulting in pAH69. The entry clone was confirmed by sequence analysis. The pAH69 was used as a template for generating plasmid pAH122, which carrying deletion on the predicted repeat domain 1, EAR motif 1, NLS, and repeat domain 2 (Δ2-1109 aa) sequence. The partial deletion of Bel2-5 was performed by inverse PCR with a specific primer set AHO85/AHO98, screened for antibiotic resistance and confirmed by PCR and sequence analysis. The pAH122 was subcloned into gateway vector pMT751 via LR reaction, resulting in pAH123. The pAH123 was transformed into MTY914 (*smt3*Δ::*KanMX4* carrying pRS415-HA-*SMT3*). | This study |
| ULP1 | Yeast MTY914 expressing the full-length sequence of yeast ULP1, *URA3* (2μ) | The full length of yeast ULP1 sequence without stop codon was amplified using a specific primer set, TKO59/TKO60 followed by amplification of *att*B-adaptor primers MTO529/MTO530 and cloned into pDONR221 by the BP reaction, resulting in pRE783. The entry clone was confirmed by sequence analysis. The pRE783 was subcloned into gateway vector pMT751 via LR reaction, resulting in pRE952. The pRE952 was transformed into MTY914 (*smt3*Δ::*KanMX4* carrying pRS415-HA-*SMT3*). | This study |
| ULP1 (C580S) | Yeast MTY914 expressing the full-length sequence of yeast ULP1 carrying an amino acid substitution on catalytic site, cysteine into serin (C580S), *URA3* (2μ) | The full length of yeast ULP1 sequence without stop codon was amplified using a specific primer set, TKO59/TKO60 followed by amplification of *att*B-adaptor primers MTO529/MTO530 and cloned into pDONR221 by the BP reaction, resulting in pRE783. The entry clone was confirmed by sequence analysis. The pER783 was used as a template for generating plasmid pRE787, which carrying an amino acid substitution on catalytic site of ULP1 (cysteine to serin, C580S) using a specific primer set, TKO83/TKO84. The substitution clone was confirmed by sequence analysis. The pER787 was subcloned into gateway vector pMT751 via LR reaction, resulting in pRE953. The pRE953 was transformed into MTY914 (*smt3*Δ::*KanMX4* carrying pRS415-HA-*SMT3*). | This study |
| Plasmid |  |  |  |
| pRK2013 | ColE1 replicon carrying RK2 transfer genes; Km^r^, *tra* |  | (Figurski and Helinski, 1979) |
| pBjGroEL4::DsRed2 | DsRed transposon delivery vector, Sm^r^, Sp^r^ |  | (Hayashi et al., 2014) |
| pUC57 | Cloning vector, Km^r^ |  | GENEWIZ, NJ, USA |
| pK18mob | Mobilizable vector for gene disruption and replacement, Mob^+^, Km^r^ |  | (Schäfer et al., 1994) |
| pDONR207 | Gateway Donor Vector, Gen^r^ |  | Thermo Fisher, MA, USA |
| pDONR221 | Gateway Donor Vector, Km^r^ |  | Thermo Fisher, MA, USA |
| pMT751 | pRS426-P_GAL1_-*att*R1-Cm^r^-ccdB-*att*R2-GFP-*CYC1* TATA |  | (Tabuchi et al., 2009) |
| pMT1271 | pRS415-*att*R1-Cm^r^-ccdB-*att*R2 |  | This study |
| Oligonucleotides for mutant constructions in *Bradyrhizobium elkanii* | | | |
| Bel2-5_EAR1-subt_R | 5’- GGCCGGGTTCGTCCATCGCCCCCGCAACCGCATCCTGCTCGTGGCGTT -3’ |  | This study |
| Bel2-5_EAR1-subt_F | 5’- AACGCCACGAGCAGGATGCGGTTGCGGGGGCGATGGACGAACCCGGCC -3’ |  | This study |
| Bel2-5_NLS-subt_R | 5’- CGGATTGTCTAGGGTCGCCGGCGCCCCCGCTGGCGCTAACTCAGCTGCATCCTGTTC -3’ |  | This study |
| Bel2-5_NLS-subt_F | 5’- GAACAGGATGCAGCTGAGTTAGCGCCAGCGGGGGCGCCGGCGACCCTAGACAATCCG -3’ |  | This study |
| Bel2-5_EAR2-subt_R | 5’- GCTGGGCCATGTCGGCTGCCTCCGCGGGGGCGTTCGCCCTTCTTGCGAGATGTG -3’ |  | This study |
| Bel2-5_EAR2-subt_F | 5’- CACATCTCGCAAGAAGGGCGAACGCCCCCGCGGAGGCAGCCGACATGGCCCAGC -3’ |  | This study |
| Bel2-5_DelRepeatI_R | 5’- CCTCGAATATCCTTGCGGAATTTCTTCCTGCGGA -3’ |  | This study |
| Bel2-5_DelRepeatI_F | 5’- TCCGCAGGAAGAAATTCCGCAAGGATATTCGAGG -3’ |  | This study |
| Bel2-5_DelRepeat2_R | 5’- TGGCCGACGCTGGAAAATTCTGTACGCCCG -3’ |  | This study |
| Bel2-5_DelRepeat2_F | 5’- CGGGCGTACAGAATTTTCCAGCGTCGGCCA -3’ |  | This study |
| pBjGroEL4:: *bel2-5*_F | 5’- GGGAACAAAAGCTGGAGCTCGGGGTGACGTGGGTGTCATTCTGCA -3’ |  | (Ratu et al., 2021) |
| pBjGroEL4:: *bel2-5* _R | 5’- GCTAGGGCGAATTGGGTACCAGTGCCAAGCTTGCATGCCTA -3’ |  | (Ratu et al., 2021) |
| Oligonucleotides for expression in yeast *Saccharomyces cerevisiae* | | | |
| Bel2-5GWF | 5’- AAAAAGCAGGCTTCGAAGGGAGATAGAACCATGGATTTCCCCTCGACCAA -3’ |  | This study |
| Bel2-5GWR1 | 5’- AGAAAGCTGGGTAACCCCTGAGTCGGTTCTGC -3’ |  | This study |
| MTO529 | 5’- GGGGACAAGTTTGTACAAAAAAGCAGGCT -3’ |  | This study |
| MTO530 | 5’-GGGGACCACTTTGTACAAGAAAGCTGGGT -3’ |  | This study |
| AHO85 | 5’- CATGGTTCTATCTCCCTTCG -3’ |  | This study |
| AHO97 | 5’- GAATTTCTTCCTGCGGAAGG -3’ |  | This study |
| AHO98 | 5’- TCGTCATCTCTCGAGCCAGT -3’ |  | This study |
| AHO99 | 5’- GACAATCCGCAAGGCCTTGC -3’ |  | This study |
| AHO100 | 5’- AATTCTGTACGCCCGTTTCC -3’ |  | This study |
| AHO101 | 5’- TGTGTCATCGAGCACCAGCC -3’ |  | This study |
| AHO102 | 5’- TTCCAGCGTCGGCCACATAC -3’ |  | This study |
| AHO103 | 5’- TAACTCAGCTGCATCCTGTT -3’ |  | This study |
| AHO104 | 5’- ATCCTGCTCGTGGCGTTCCG -3’ |  | This study |
| JHO18 | 5’- TACCCAGCTTTCTTGTACAA -3’ |  | This study |
| TKO59 | 5’- AAAAAGCAGGCTTCACCATGTCAGTTGAAGTAGATAA -3’ |  | This study |
| TKO60 | 5’- AGAAAGCTGGGTATTTTAAAGCGTCGGTTAA -3’ |  | This study |
| TKO83 | 5’- CCAAATGGCTACGACAGTGG -3’ |  | This study |
| TKO84 | 5’- TTGCTGCGGACAATCTAAAT -3’ |  | This study |
| MTO1154 | 5’- ATGTACCCATACGATGTTCCTGACTATGCGTCGGACTCAGAAGTCAA -3’ |  | This study |
| MTO1155 | 5’- CGCTCGTGTATTTATTTGTA -3’ |  | This study |
| MTO1156 | 5’- AAAAAGCAGGCTAAACGACACTGCACAACCCG -3’ |  | This study |
| MTO1157 | 5’- AGAAAGCTGGGTCATCAGCTGGGAAAGAGGCG -3’ |  | This study |
| Oligonucleotides for qRT-PCR | | | |
| NINa-F | 5'- TAACATGCGATGCTGATCTTG -3' |  | (Yasuda et al., 2016) |
| NINa-R | 5'- TGATTTAGAGGCGAAGCTTGA -3' |  | (Yasuda et al., 2016) |
| ENOD40-F | 5'- GAAAGGGGTGTGAGAGGAGAG -3' |  | (Yasuda et al., 2016) |
| ENOD40-R | 5'- CGCCACTCAAGAAAGAATGTT -3' |  | (Yasuda et al., 2016) |
| SUBI-F | 5'- AGCTATTCGCAGTTCCCAAAT -3' |  | (Yasuda et al., 2016) |
| SUBI-R | 5'- CAGAGACGAACCTTGAGGAGA -3' |  | (Yasuda et al., 2016) |
| PR1-F | 5'- AGTGGCACAGATGCAGTGAA -3' |  | (Yasuda et al., 2016) |
| PR1-R | 5'- CATGTCACTTTGGCACATCC -3' |  | (Yasuda et al., 2016) |
| PDF1-F | 5'- CATACAGGGGTCCATGCTTC -3' |  | (Yasuda et al., 2016) |
| PDF1-R | 5'- GCATCTGCCTCTGAGCAAGT -3' |  | (Yasuda et al., 2016) |
| Rj4-F | 5'- CAAGTTCGACTAAAAAGGCATT -3' |  | (Yasuda et al., 2016) |
| Rj4-R | 5'- TCTGGTTGAATACGAACCCAT -3' |  | (Yasuda et al., 2016) |
| ACO1-F | 5’- ACTTGGAGAAGCTCAGTGGTGA -3’ |  | (Ratu et al., 2021) |
| ACO1-R | 5’- CACAGTGTCCAATATGTCATGAGGA -3’ |  | (Ratu et al., 2021) |
| WRKY33-F | 5’- GAAACACTTCAACTGAGGGTCAACA -3’ |  | (Ratu et al., 2021) |
| WRKY33-R | 5’- GTTTGCTTTGCAGCTTCACTGG -3’ |  | (Ratu et al., 2021) |
| ERF1b-F | 5’- TGGATGCTCTCCCGTTGTGG -3’ | XM_003535756.4 PREDICTED: Glycine max ethylene-responsive transcription factor 1B (LOC100785936), mRNA | This study |
| ERF1b-R | 5’- ATCAGCCCCAAGGTCATGGA -3’ |  | This study |

^a^Pol^r^, polymyxin resistant; Km^r^ , kanamycin resistant; Sm^r^ , streptomycin resistant; and Sp^r^ , spectinomycin resistant.

^b^United States Department of Agriculture, Beltsville, MD.

**References**

Devine T. E., and O`Neill, J. J. (1986). Registration of BARC-2 (*Rj4*) and BARC-3 (*rj4*) soybean germplasm. Crop Sci*.* 26, 1263–1264. doi:10.2135/cropsci1986.0011183X002600060055x.

Figurski, D. H., and Helinski, D. R. (1979). Replication of an origin-containing derivative of plasmid RK2 dependent on a plasmid function provided in trans (plasmid replication/replication origin/trans-complementation/broad host range/gene cloning). Proc. Nati. Acad. Sc 76, 1648–1652. doi:10.1073/pnas.76.4.1648.

Francisco, P. B., and Akao, S. (1993). Autoregulation and nitrate inhibition of nodule formation in soybean cv. Enrei and its nodulation mutants. J. Exp. Bot. 44, 547–553. doi:10.1093/jxb/44.3.547.

Hayashi, M., Shiro, S., Kanamori, H., Mori-Hosokawa, S., Sasaki-Yamagata, H., Sayama, T., et al. (2014). A thaumatin-like protein, *Rj4*, controls nodule symbiotic specificity in soybean. Plant Cell Physiol. 55, 1679–1689. doi:10.1093/pcp/pcu099.

Ikeda, S., Rallos, L. E. E., Okubo, T., Eda, S., Inaba, S., Mitsui, H., et al. (2008). Microbial community analysis of field-grown soybeans with different nodulation phenotypes. Appl. Environ. Microbiol. 74, 5704–5709. doi:10.1128/AEM.00833-08.

Ratu, S. T. N., Teulet, A., Miwa, H., Masuda, S., Nguyen, H. P., Yasuda, M., et al. (2021). Rhizobia use a pathogenic-like effector to hijack leguminous nodulation signalling. Sci. Rep. 11, 1–15. doi:10.1038/s41598-021-81598-6.

Schäfer, A., Tauch, A., Jäger, W., Kalinowski, J., Thierbach, G., and Pühler, A. (1994). Small mobilizable multi-purpose cloning vectors derived from the *Escherichia coli* plasmids pK18 and pK19: selection of defined deletions in the chromosome of *Corynebacterium glutamicum*. Gene 145, 69-73. doi:10.1016/0378-1119(94)90324-7.

Simon, R., Priefer, U., and Pühler, A. (1983). A broad host range mobilization system for *in vivo* genetic engineering: transposon mutagenesis in gram negative bacteria. Biotechnology, 784–790. doi:10.1038/nbt1183-784.

Tabuchi, M., Kawai, Y., Nishie-Fujita, M., Akada, R., Izumi, T., Yanatori, I., et al. (2009). Development of a novel functional high-throughput screening system for pathogen effectors in the yeast *Saccharomyces cerevisiae*. Biosci. Biotechnol. Biochem*.* 73, 2261–2267. doi:10.1271/bbb.90360.

Yasuda, M., Miwa, H., Masuda, S., Takebayashi, Y., Sakakibara, H., and Okazaki, S. (2016). Effector-triggered immunity determines host genotype-specific incompatibility in legume-*rhizobium* symbiosis. Plant Cell Physiol. 57, 1791–1800. doi:10.1093/pcp/pcw104.
